# Targeting the DYRK1A kinase prevents cancer progression and metastasis and promotes cancer cells response to G1/S targeting chemotherapy drugs

**DOI:** 10.1038/s41698-024-00614-w

**Published:** 2024-06-05

**Authors:** Amina Jamal Laham, Raafat El-Awady, Maha Saber-Ayad, Ni Wang, Gang Yan, Julien Boudreault, Suhad Ali, Jean-Jacques Lebrun

**Affiliations:** 1 Department of Medicine, Cancer Research Program, McGill University Health Center, Montreal, Quebec H4A 3J1 Canada; 2 College of Medicine, University of Sharjah, Sharjah, 27272 United Arab Emirates; 3 Research Institute for Medical and Health Sciences, University of Sharjah, Sharjah, 27272 United Arab Emirates; 4 College of Pharmacy, University of Sharjah, Sharjah, 27272 United Arab Emirates

**Keywords:** Breast cancer, Colon cancer

## Abstract

Metastatic cancer remains incurable as patients eventually loose sensitivity to targeted therapies and chemotherapies, further leading to poor clinical outcome. Thus, there is a clear medical gap and urgent need to develop efficient and improved targeted therapies for cancer patients. In this study, we investigated the role of DYRK1A kinase in regulating cancer progression and evaluated the therapeutic potential of DYRK1A inhibition in invasive solid tumors, including colon and triple-negative breast cancers. We uncovered new roles played by the DYRK1A kinase. We found that blocking DYRK1A gene expression or pharmacological inhibition of its kinase activity via harmine efficiently blocked primary tumor formation and the metastatic tumor spread in preclinical models of breast and colon cancers. Further assessing the underlying molecular mechanisms, we found that DYRK1A inhibition resulted in increased expression of the G1/S cell cycle regulators while decreasing expression of the G2/M regulators. Combined, these effects release cancer cells from quiescence, leading to their accumulation in G1/S and further delaying/preventing their progression toward G2/M, ultimately leading to growth arrest and tumor growth inhibition. Furthermore, we show that accumulation of cancer cells in G1/S upon DYRK1A inhibition led to significant potentiation of G1/S targeting chemotherapy drug responses in vitro and in vivo. This study underscores the potential for developing novel DYRK1A-targeting therapies in colon and breast cancers and, at the same time, further defines DYRK1A pharmacological inhibition as a viable and powerful combinatorial treatment approach for improving G1/S targeting chemotherapy drugs treatments in solid tumors.

## Introduction

Cell cycle is one of the main pathways to be dysregulated in tumorigenesis and cancer progression. As such targeting cell cycle remains a favored approach for developing anti-cancer therapeutics^1^. Protein phosphorylation has been implicated in carcinogenesis by regulating many cellular processes such as proliferation, apoptosis, differentiation, and metabolism^2^. Because protein kinases are easily druggable, extensive efforts have been spent to explore their potential as targeted therapy in various types of cancer and in fact, many current anti-cancer drugs and treatments rely upon protein kinase inhibition^3^. The dual-specificity tyrosine phosphorylation-regulated kinases (DYRKs) can auto-phosphorylate their activation loop on tyrosine residue while phosphorylating their specific substrates on threonine and serine residues^4^. Of all the 7 DYRK family members, DYRK1A has been the most extensively studied^5^. The DYRK1A gene maps to human chromosome 21 within the Down syndrome critical region (DSCR) and its overexpression has been implicated in neuronal development deficits and brain abnormalities in Down syndrome^6^. DYRK1A regulates cell cycle and differentiation of neuronal cells by inducing G0/G1 arrest through phosphorylation and subsequent degradation of cyclin D as well as through stabilization of the cyclin-dependent kinase inhibitor p27 Kip protein^7^. While DYRK1A affects the cell cycle state of neurons and plays an essential role in neurogenesis^8^, it was also found to regulate cyclin-dependent kinase-1 (CDK1) activity in glioblastoma cells and DYRK1A inhibition exhibit anti-tumor effects in glioblastoma^9,10^. The therapeutic use of DYRK1A-mediated cell cycle regulation was recently extended to other diseases, such as diabetes and myocardial infarction, as DYRK1A inhibition was found to sustain pancreatic beta cells and cardiomyocytes growth^11–16^. Despite having important cell cycle regulatory functions, a role for DYRK1A in cancer, other than glioblastoma remains to be fully investigated. Some studies reported DYRK1A to exert antitumorigenic effects while others suggested it can promote tumorigenesis^17–20^. Interestingly, we recently found that expression of DYRK1A is associated with bad prognosis and poor clinical outcome in colorectal cancer patients, strongly suggesting a role for this kinase in colon cancer formation and/or progression^21^.

In this study, we explored the role of DYRK1A in solid tumor models, including colorectal and triple negative breast cancer (TNBC). We found that DYRK1A is essential for keeping colon and breast cancer cells in the G0 quiescence state. We further found that inhibition of DYRK1A activity or expression decreased cell growth in vitro and reduced tumorigenesis and metastasis in vivo, using preclinical models of colon and breast cancers. We also show that releasing cancer cells from their quiescent state through inhibition of DYRK1A activity or expression resulted in cancer cells accumulation in G1/S phase, further delaying their progression to the G2/M phase, consistent with reduced cell growth. Building on these findings, we further found that blocking DYRK1A expression or activity enhanced chemotherapy drugs responses when using cell cycle G1/S phase-specific drugs (cisplatin, topotecan) but not drugs targeting the M phase (paclitaxel). These effects were demonstrated in both colon cancer and breast cancer in vitro and in vivo. Altogether, our results define DYRK1A as an important therapeutic target for both colon cancer and triple negative breast cancer and highlight DYRK1A pharmacological inhibition as a potential new therapeutic approach for these cancer patients. This study also underscores a new potential adjuvant therapeutic strategy for these solid tumors and defines DYRK1A pharmacological inhibition as a potent combinatorial treatment approach to improve G1/S targeting chemotherapy drug efficacy.

## Results

### DYRK1A is highly expressed in agressive breast tumors and correlates with poor prognosis

**Fig. 1:**
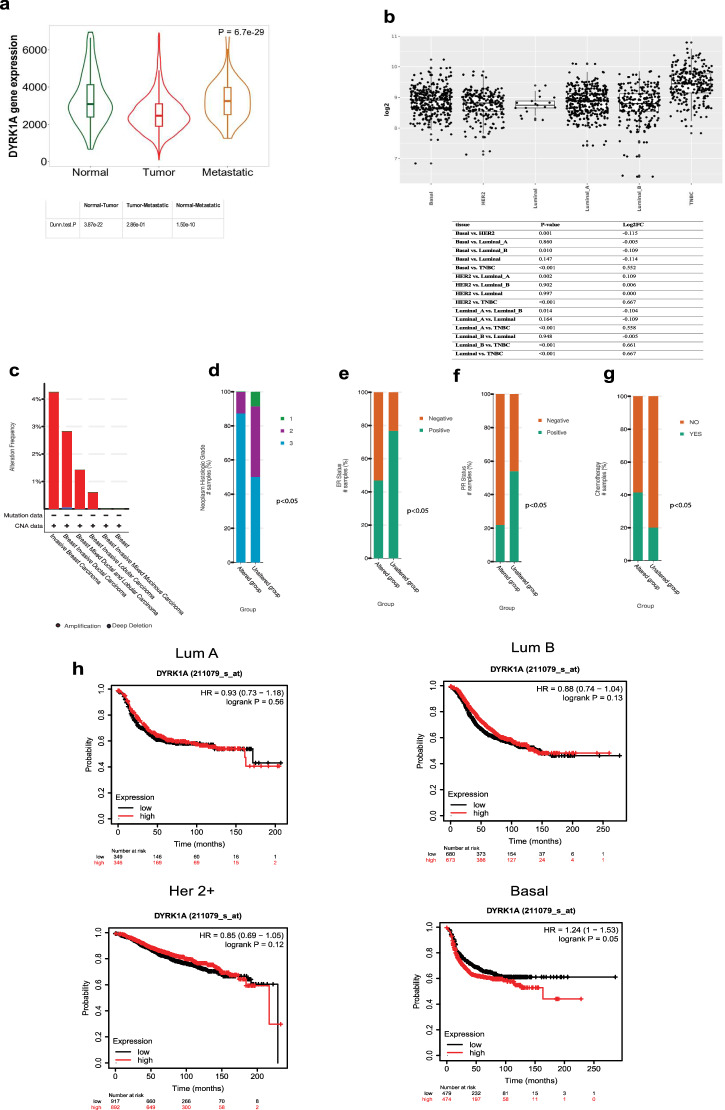
DYRK1A is highly expressed in aggressive breast tumors and correlates with poor prognosis. **a** A violin blot showing DYRK1A expression in normal, tumor, and metastatic breast tissues generated from the TNMplot tool. **b** Upper panel: DYRK1A expression level in breast cancer molecular subtypes generated using GENT2 web-based tool representing data from NCBI GEO database. Lower panel: Table showing the statistical analysis generated from GENT2 web-based tool. **c**–**g** Bar blots showing (**c**) amplification mutations, (**d**) histologic grade, (**e**) ER status, (**f**) PR status, and (**g**) chemotherapy samples for DYRK1A altered group (amplified) in breast cancer (METABRIC, Nature 2012 & Nat Commun 2016) dataset generated using cBioPortal. *P*-value generated from the cBioPortal tool. RFS was generated using the Kmplot tool. We chose DYRK1A (211079_s_at) Affymetrix ID, we ran RFS analysis. **a** luminal A, luminal B, HER2 + and Basel. *P*-value generated from the Kmplot tool.

The role of DYRK1A in cancer is not well studied and remains controversial. While some studies reported DYRK1A to exert antitumor activities, others suggested that it may exhibit pro-tumoral activity^17–20^. We recently showed that DYRK1A is significantly upregulated in late stages (IIIA to IVB) colorectal tumors and that its high expression correlates to a very poor prognosis for colon cancer patients, suggesting a role in promoting tumorigenesis in these tumors^21^. In other types of solid tumors, such as breast cancer, the role of DYRK1A remains unclear. While DYRK1A expression level seems decreased in breast cancer tumor samples^22^, DYRK1A knockdown or inhibition of its activity reduced breast cancer cell proliferation^23^. Thus, DYRK1A role in breast tumor formation and progression may be context dependent and/or subtype specific and clearly needs further investigation. To start address this, we examined DYRK1A expression in the different molecular subtypes of breast cancer. First, we found DYRK1A expression to be significantly upregulated in metastatic breast cancer, compared to normal breast tissues (Fig. 1a). Interestingly, we further found DYRK1A expression to be highly upregulated in triple negative breast cancer (TNBC), the most aggressive form of the disease, compared to other breast cancer molecular subtypes (Fig. 1b). We further assessed the DYRK1A status in breast cancer using the Breast Cancer (METABRIC, Nature 2012 & Nat Commun 2016) data set from cBioportal. While DYRK1A has a low mutational count in breast cancer (only 2.5% amplification mutations), these patients (altered group) mostly exhibit aggressive tumors (histological grade 2 and 3) with ER-/PR- status and are more likely categorized as patients who received chemotherapy (Fig. 1c–g). Furthermore, and consistent with these data, we also found, using Kaplan–Meier analysis, that high DYRK1A expression significantly correlates with lower relapse-free survival in the basal subtype, which comprises all TNBC patients, but not in luminal and HER2+ tumors (Fig. 1h). These results indicate that DYRK1A differential expression and association with poor prognosis is specifically linked to the TNBC molecular subtype and strongly suggest that DYRK1A is implicated in TNBC patient tumorigenesis.

### DYRK1A inhibition blocks colon and breast cancer tumor growth in vitro and in vivo

**Fig. 2:**
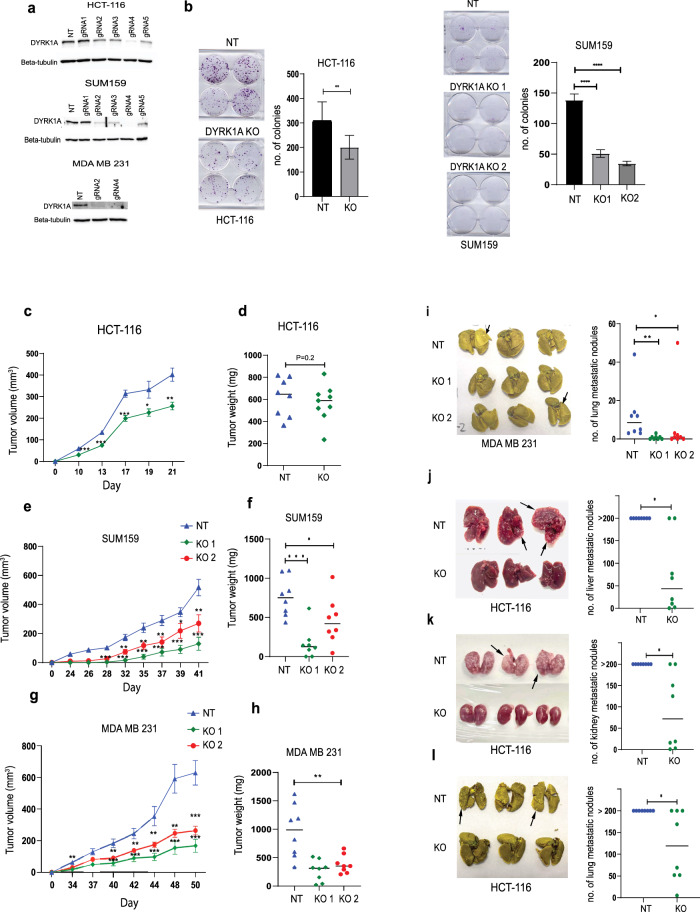
DYRK1A inhibition blocks colon and breast cancer tumor growth in vitro and in vivo. **a** Western blots showing DYRK1A expression in HCT-116, SUM-159 and MDA-MB-231cells silenced or not with various CRISPR gRNAs. **b** Clonogenic assay for DYRK1A-KO and NT-KO HCT-116 and SUM-159 cells. Colonies stained via crystal violet and quantified through manual count. **c** Subcutaneous transplantation of DYRK1A-KO and NT-KO HCT-116 cells in the right flank of NSG mice (*n* = 8). Tumor volumes were measured every 2 days. **d** HCT-116 ex-in vivo tumor weights at experimental endpoint. **e, g** orthotopic transplantation of DYRK1A-KOs and NT-KO SUM-159 and MDA-MB-231cells in the mammary fat pad of NSG mice. Tumor volumes were measured every 2 days. **f**, **h** SUM-159 and MDA-MB-231 ex-in vivo tumor weights at experimental endpoint. Error bars represent ±SEM of three independent experiments or for *n* = 8. * Represent the *p-*value (*p < 0.05, **p < 0.01, ***p < 0.001, ****p < 0.0001) generated using two-sided *T*-test. **i** Left panel: Representative image showing the spontaneous MDA MB 231 lung nodules after fixation. Right panel: Dot plot representing counted lung nodules after fixation with Bouin’s solution. Error bars represent ±SEM of *n* = 8, dot blot middle line represents the median. * Represent the *p*-value (*p < 0.05, **p < 0.01, ***p < 0.001, ****p < 0.0001) generated using Mann–Whitney *U-*test (*n* = 8). **j**–**l** DYRK1A KO and NT-KO HCT-116 cells injected intravenously into the tail vein of NSG male mice (*n* = 8). Left panels: Representative images showing liver, kidney, and lung metastatic nodules. Right panels: dot plot representing counted nodules. The middle line represents the median (*n* = 8). * Represent the *p*-value (*p < 0.05, **p < 0.01, ***p < 0.001, ****p < 0.0001) generated using Mann–Whitney *U*-test (*n* = 8).

To further assess the DYRK1A effects on both colorectal and TNBC cell proliferation at the molecular level, we blocked DYRK1A gene expression by means of CRISPR-based gene editing, as previously described^24,25^. For this, we generated DYRK1A gene knockouts (KOs) in HCT-116 colon cancer cells, SUM-159 and MDA-MB-231 TNBC cells. As shown in (Fig. 2a) five specific gRNAs were tested in all cell lines and gRNAs 2 and 4 were selected for all further experiments, as showing near complete DYRK1A gene silencing. Non targeting (NT) gRNAs were used as negative controls. Cell proliferation and tumorigenic potential of the parental and gene edited KO cell lines were then assessed using the clonogenic assay. Interestingly, we found that DYRK1A KO led to a significant inhibition of cancer cell proliferation, compared to NT-KOs, in both colon cancer and TNBC cells (Fig. 2b).

To address how these findings translate to tumor formation in vivo, we used preclinical models of colon and breast cancer tumorigenesis, as previously shown^24,25^. Briefly, immunodeficient NSG mice were injected subcutaneously with DYRK1A-KO or NT-KO colon cancer HCT-116 cells and tumor volume was monitored daily using caliper measurements. At experimental endpoint, tumors were resected and weighted. For the TNBC preclinical model, DYRK1A-KO or NT-KO SUM-159 and MDA-MB-231 cells were orthotopically transplanted using a mammary fat pad injection xenograft model^24,25^. Interestingly, as shown in (Fig. 2c, d), blocking DYRK1A gene expression in colon cancer significantly reduced the tumor volume over time as well as the tumor weight at experimental end point. The effects of DYRK1A gene silencing were even more pronounced in TNBC, showing a strong reduction in both tumor volume and tumor weight at experimental endpoint (Fig. 2e, h). These results are consistent with what was observed in vitro and indicate that DYRK1A gene silencing can efficiently prevent or delay tumor formation.

MDA-MB-231 cells tend to spontaneously form lung metastasis when transplanted in NSG mice. Interestingly, as shown in (Fig. 2i), suppressing DYRK1A gene expression strongly and significantly reduced the number of metastatic lung nodules, suggesting that DYRK1A plays an important role during the metastatic process in breast cancer. To further address this, we analyzed the protein expression levels of various EMT markers in the resected tumors from the NT and DYRK1A KO tumor samples from the MDA-MB-231 xenografts. While no significant changes were observed with E-cadherin, N-cadherin or beta-catenin (data not shown), we found a potent and significant decrease in the expression of 3 of the main mesenchymal markers, vimentin, matrix metalloproteinases-9 (MPP-9) and snail (Supplementary Fig. 1), suggesting that DYRK1A affects the EMT phenotype during the metastatic process.

Late tumor stage colorectal cancer commonly metastasizes to the liver, lungs and kidneys^26^. To assess whether DYRK1A KO also affect colon cancer progression and metastasis, we used an experimental metastasis preclinical model^27^. Briefly, DYRK1A-KO and control NT-KO HCT-116 cells were administered to NSG mice through intravenous tail vein injection. Tumors were allowed to growth for 21 days before resection of the liver, kidneys, and lungs. Interestingly, as shown in (Fig. 2g–i), DYRK1A gene silencing had profound and significant effects in reducing the number of metastatic nodules in all tissues examined (liver, kidneys, and lungs). Combined, these results highlight DYRK1A as a central regulator of both primary tumor formation and metastatic spread to distant organs. Furthermore, they also indicate that DYRK1A inhibition potently decreases tumor growth and efficiently prevents the formation of liver, kidney, and lung metastatic nodules.

### DYRK1A expression decreases during cell cycle progression

**Fig. 3:**
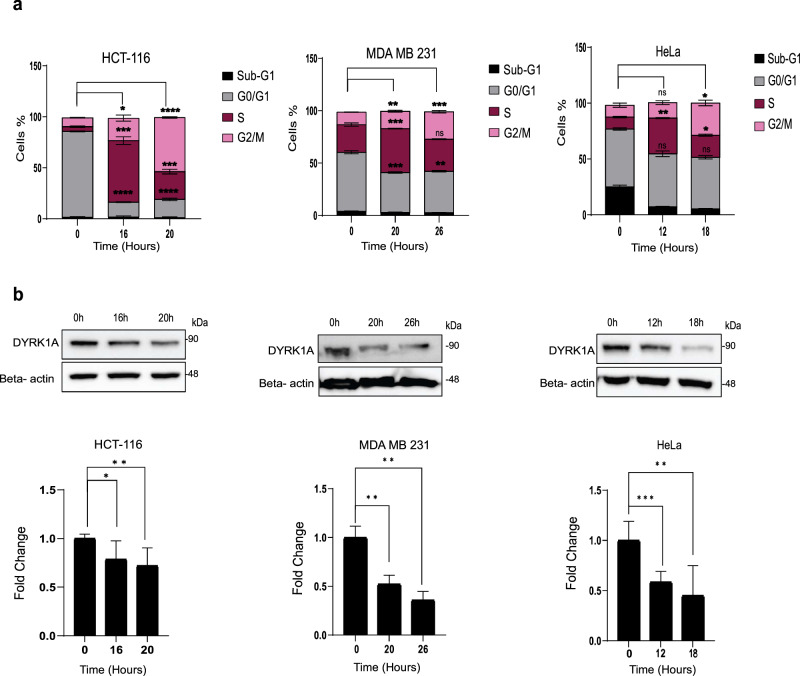
DYRK1A expression decreases during cell cycle progression. **a** Cell cycle analysis using PI staining for HCT-116, MDA MB 231, and HeLa cells. Cells were synchronized by contact inhibition and serum starvation and subsequently released into the cell cycle through serum addition. **b** Western blots showing DYRK1A expression in each cell cycle phase. lower panels: Quantification of DYRK1A protein expression using beta-actin as a loading control. Error bars represent ±SEM of three independent experiments. * Represent the *p*-value compared to control (*p < 0.05, **p < 0.01, ***p < 0.001, ****p < 0.0001) generated using two-sided *T*-test.

Previous studies have shown that DYRK1A induces quiescence and G1 arrest in neuronal cells^28^ and in normal fibroblasts^29^. To address the mechanisms by which DYRK1A regulates tumorigenesis, we first examined whether DYRK1A expression levels varied during cell cycle progression. Cell cycle analysis was carried out using propidium iodide (PI) staining which allows gating for sub-G1, G0/G1, S and G2/M phases. Cancer cells of different origins, including colorectal (HCT-116), TNBC (MDA-MB-231), and cervical (HeLa) were first synchronized at G0/G1 border through serum starvation before being allowed to re-enter cell cycle progression through serum addition. As shown in (Supplementary Fig. 2a–c), all 3 cell lines are redistributed in the different phases of the cell cycle in a cell-specific and time-dependent manner. As shown in (Fig. 3), optimal time points representing each phase of the cell cycle (G0/G1, S and G2/M) were then selected for each cell line (Fig. 3a) to further assess DYRK1A protein expression levels (Fig. 3b). The sub-G1 condition which reflects DNA fragmentation and is indicative of cell death was not retained, as very few cells were found in sub-G1. Interestingly, DYRK1A protein levels were maximum in cancer cells in G0/G1 and significantly decreased as cells progressed throughout the S and G2/M phases of the cell cycle. These results show that DYRK1A expression levels vary during cell cycle, suggesting that DYRK1A may play an important role in cell cycle progression and are also consistent with a previous report showing that DYRK1A could affect the DREAM complex assembly, thereby promoting cell quiescence^28^.

### Blocking DYRK1A relieves cancer cells from quiescence, prolonges G1/S and delays G2/M cell cycle entry

**Fig. 4:**
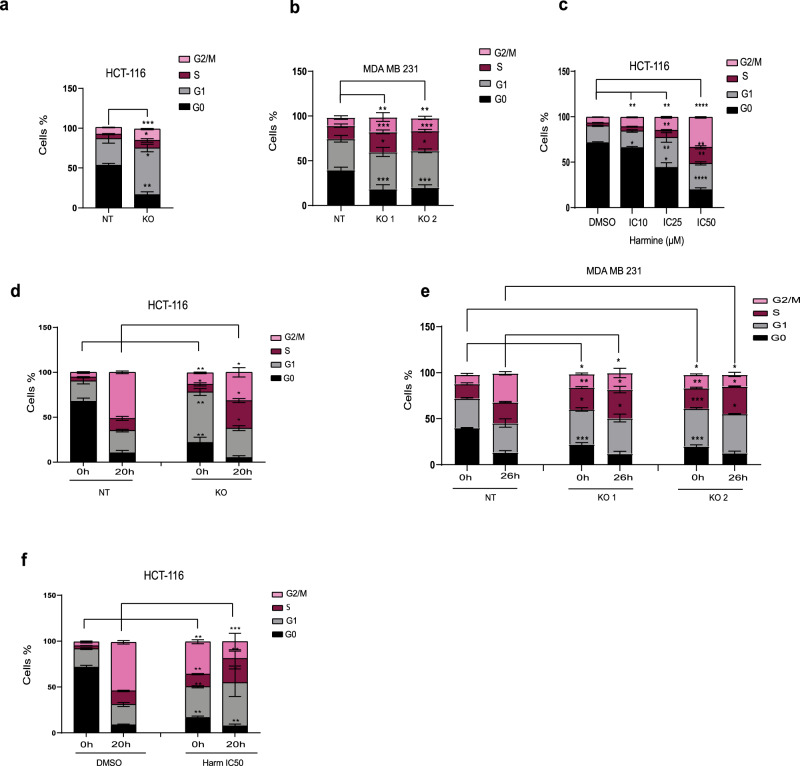
Blocking DYRK1A relieves cancer cells from quiescence, prolonges G1/S and delays G2/M cell cycle entry. Cell cycle analysis with Pyronin Y/Hoechst 33342 double staining for DYRK1A KO and NT-KO in (**a**) HCT-116 and (**b**) MDA-MB-231 cells, serum-starved for 24 h. **c** Cell cycle analysis with Pyronin Y/Hoechst 33342 double staining for HCT-116 treated with various concentrations of harmine, as indicated. Pyronin Y/Hoechst 33342 staining, following serum starvation and release into cell cycle for DYRK1A KO and NT-KO in (**d**) HCT-116, (**e**) MDA-MB-231 cells and (**f**) HCT-116 cells treated with harmine IC50. Error bars represent ±SEM of three independent experiments. * Represent the *p*-value compared to control (DMSO 0%FBS or NT 0%FBS) (*p < 0.05, **p < 0.01, ***p < 0.001, ****p < 0.0001) generated using two-sided *T*-test.

To further address the role DYRK1A on cell quiescence and cell cycle progression, we performed deeper cell cycle analysis, using PyroninY/Hoechst 33342 double staining to visualize, gate and quantify cells in G0 alone, G1, S and G2/M, as described previously^30^. DYRK1A-KO and NT-KO control colon cancer (HCT-116) and breast cancer (MDA-MB-231) cells were serum starved for 24 h to induce quiescence before being processed with the PyroninY/Hoechst 33342 double staining. As shown in (Fig. 4a, b), DYRK1A gene silencing strongly reduced the percentage of quiescent cancer cells (G0), while promoting cancer cells progression through the cell cycle in both colon and TNBC cells.

To assess whether DYRK1A kinase activity was required for the mediation of these effects, we used a pharmacological inhibition approach, using the DYRK1A inhibitor, harmine at various concentrations (IC10, IC25 and IC50). As shown in (Fig. 4c), the number of HCT-116 quiescent cells significantly decreased, when using the DYRK1A pharmacological inhibitor, while the numbers of cells cycling through G1, S and G2/M phases concomitantly increased, in a concentration-dependent manner. These results are consistent with those observed with the DYRK1A KOs and suggest that DYRK1A kinase activity regulates the cell cycle primarily through maintaining cancer cells in the quiescent state. Original flow cytometry data, gating and values are shown in (Supplementary Fig. 3).

As shown in this study, DYRK1A KO leads to inhibition of colony formation in vitro (Fig. 2a) as well as inhibition of tumor formation in vivo in preclinical models of breast and colon cancers (Fig. 2c–h). At the same time, we also found that blocking DYRK1A expression or activity releases cells from quiescence, enforcing cancer cells to enter G1/S (Fig. 4a–c). This suggests that inhibition of DYRK1A could decrease quiescence and increase the number of cells in the G1 and S phases of the cell cycle but prevent or delay entry to the M phase, thereby ultimately inhibiting cell proliferation. To test this hypothesis, colon cancer (HCT-116) and TNBC (MDA-MB-231) DYRK1A-KO or NT control cells were synchronized and subsequently allowed to renter cell cycle. As shown in (Fig. 4d), serum-induced cell cycle progression in control NT colon cancer cells led to an expected release from G0 with concomitant increase in G1/S and G2/M cell numbers with 50% of the cells reaching G2/M by 20 h. Consistent with what is shown in (Fig. 4a), DYRK1A gene silencing led to decreased G0 and increased G1 cell numbers, compared to NT control cells, under cell cycle arrest (starvation condition; time 0). Interestingly, however, serum-induced cell cycle progression in the DYRK1A KO cells led to a stronger increase in percent of cells in the G1/S phase while showing a significant decrease in the percent of cells in G2/M phase, compared to control NT cells. The exact same effects and profiles were obtained when assessing the effects of DYRK1A gene knockout in TNBC cell line (MDA-MB-231) (Fig. 4d). Altogether, these results indicate that blocking DYRK1A expression releases cells from quiescence to enter G1/S but prevents or delays entry to the G2/M phase of the cell cycle in both colon and breast cancer cells. To address the role of DYRK1A kinase activity in the mediation of these effects, we used a pharmacological inhibition approach with the harmine inhibitor. As shown in (Fig. 4f), while most control cells (DMSO) entered the G2/M phase by 20 h, cells treated with harmine (IC50 dose) exhibited a strong increase in the percent of cells in G1/S with a significant decrease in the percent of cells in G2/M compared to untreated cells, consistent with our results obtained with the DYRK1A CRISPR-KOs.

### Blocking DYRK1A differrentially regulates expression of G1/S and G2/M regulators

**Fig. 5:**
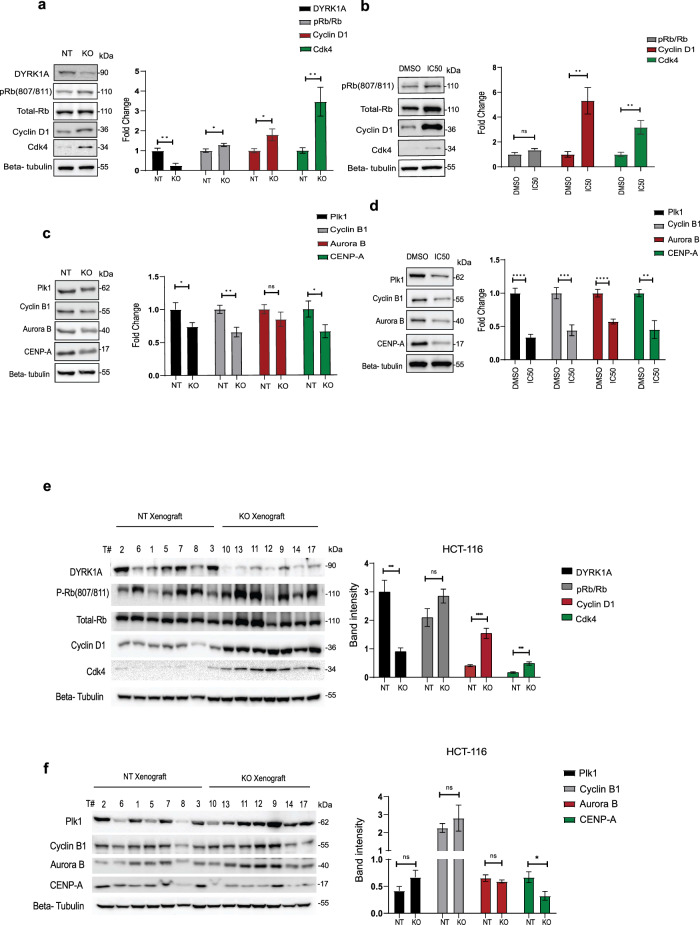
Blocking DYRK1A differentially regulates expression of G1/S and G2/M regulators. Right panels: Western blot analysis for the expression of various G1/S cell cycle regulators in (**a**) DYRK1A-KO, NT HCT-116 cells and (**b**) HCT-116 treated with harmine at IC50. Left panels: Protein expression quantification normalized to beta -tubulin. Western blot analysis for the expression of various G2/M cell cycle regulators in (**c**) DYRK1A-KO, NT HCT-116 cells and (**d**) HCT-116 treated with harmine at IC50. Left panels: Protein expression quantification normalized to beta -tubulin. Left panels: Western blot analysis for various (**e**) G1/S and (**f**) G2/M phase cell cycle regulators expression in DYRK1A-KO and NT HCT-116 tumor xenografts. Right panels: Protein expression quantification normalized to beta -tubulin. Error bars represent ±SEM of three independent experiments. * Represent the *p*-value (*p < 0.05, **p < 0.01, ***p < 0.001, ****p < 0.0001) generated using two-sided *T*-test.

To further address the molecular mechanisms by which DYRK1A regulates cell cycle progression, we examined the effects of blocking DYRK1A expression or activity on the expression levels and activation states of the main cell cycle regulatory proteins. As shown in (Fig. 5a) DYRK1A KO led to increased Rb phosphorylation, cyclin D1 and CDK4 expression, consistent with a progression through G1 to S phase of the cell cycle. Interestingly, we observed the same exact profiles when cells were treated with the DYRK1A inhibitor, harmine at IC50 concentration. Convincingly, all these effects are concentration-dependent (Fig. 5b). Collectively these results show that depletion/inhibition of DYRK1A leads to increased expression of G1/S cell cycle activators, further releasing cancer cells from quiescence and promoting their progression into cell cycle.

To further address the molecular mechanisms by which DYRK1A depletion delay G2/M entry, we examined the effects of blocking DYRK1A expression or activity on the expression levels of various cell cycle regulatory proteins acting in G2/M. As shown in (Fig. 5c) DYRK1A KO led to significant decrease in the expression of the G2/M regulators, including cyclin B1, mitotic proteins that are essential for mitotic chromosome segregation (Plk1, Aurora B) and the centromere protein A (CENP-A), all consistent with a delay in G2/M phase entry. The exact same effects were observed when cells were treated with the DYRK1A inhibitor harmine, showing significant decrease in the expression of the G2/M regulatory proteins (Fig. 5d)

Finally, we also assessed expression levels of the various G1/S and G2/M regulators in the resected tumor samples from the xenograft experiments performed in Fig. 2. Interestingly, G1/S expression levels were significantly higher in tumors resected from the DYRK1A KO animals compared to NT control tumors, highly consistent with what observed in vitro with the DYRK1A KO and harmine treated colon cancer cells (Fig. 5e) When assessing expression levels of the G2/M regulators, no significant changes were observed in the cyclin B1, PLK1 and aurora B levels, possibly due to the low numbers of animal used in these preclinical experiments. However, we found a consistent and significant decrease in expression of the centromere protein A (CENP-A), consistent with the in vitro data, highlighting CENPA as a main and critical G2/M target for DYRK1A and confirming that DYRK1A inhibition decreases expression level of critical G2/M regulators in vitro and in vivo (Fig. 5f)

Altogether, these results support our hypothesis that DYRK1A gene silencing and/or pharmacological inhibition decrease cell quiescence, leading to accumulation of cancer cells in G1/S, preventing/delaying cells to enter G2/M. These results are also consistent with our biological readout data showing a decrease in cell growth/colony formation in vitro and inhibition of tumor growth in vivo, in the absence of DYRK1A (Fig. 2)

### Inhibition/depletion of DYRK1A sensitizes cancer cells to G1/S-targeting chemotherapy drugs in vitro and in vivo

**Fig. 6:**
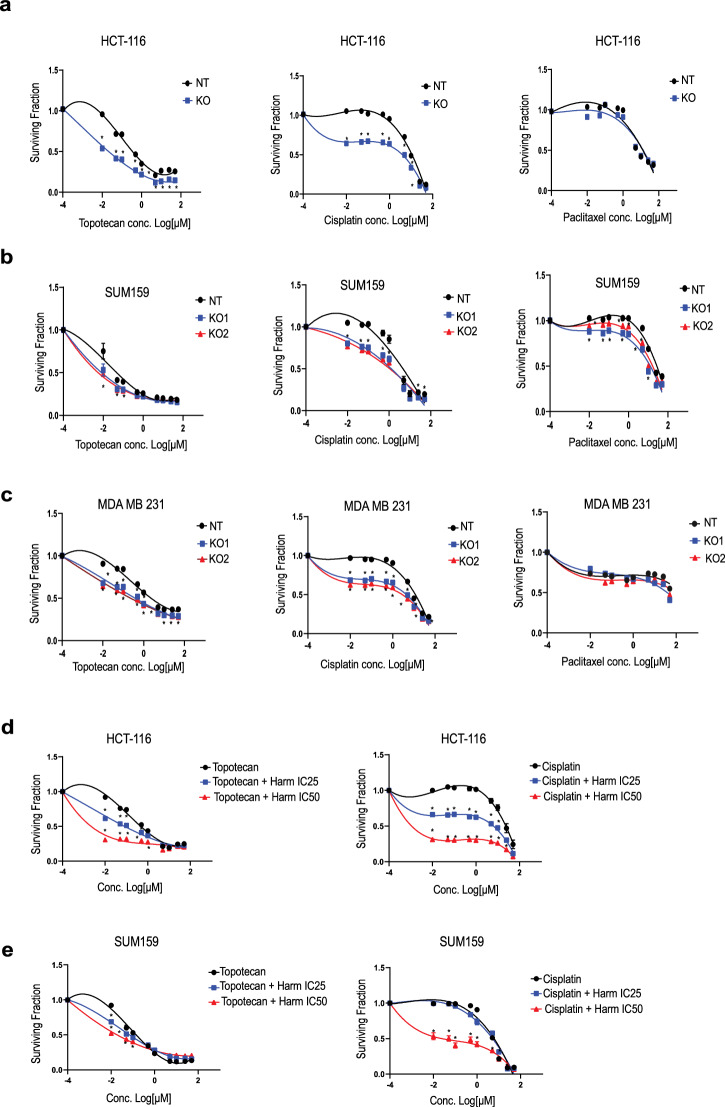
Inhibition/depletion of DYRK1A sensitizes cancer cells to G1/S phase-targeting chemotherapy drugs in vitro*.* Dose-response curves after various concentrations of chemotherapeutic drugs (topotecan, cisplatin and paclitaxel, 48 h treatments) in DYRK1A-KOs and NT-KOs for (**a**) HCT-116, (**b**) SUM-159 and (**c**) MDA-MB-231 cells or parental HCT-116 (**d**) and MDA-MB-231 (**e**) cells co-treated with harmine (IC25 and IC50). Error bars represent ±SEM of three independent experiments. * Represent the *p*-value (*p < 0.05, **p < 0.01, ***p < 0.001, ****p < 0.0001) generated using two-sided *T*-test.

Having shown that DYRK1A inhibition prolongate G1/S phase of the cell cycle, this suggests that DYRK1A inhibition could sensitize cancer cells to chemotherapy drugs that target cells in G1/S phase. To address this, we first assessed cell cytotoxicity by SRB assay in control (NT) and DYRK1A-KO colon (HCT-116) and TNBC (SUM159, MDA-MB-231) cancer cells using 2 different chemotherapy drugs that target cells in the G1/S phase, topotecan and cisplatin^31,32^. Topotecan targets topoisomerase 1 while cisplatin can induce DNA inter or intra-strand crosslinks^31–34^. As a control and to further demonstrate our hypothesis, we also used paclitaxel, another chemotherapy drug that acts outside G1/S, by targeting cells undergoing mitosis at the M phase^35^. As shown in (Fig. 6a), all 3 chemotherapy drugs efficiently reduced the survival of the colon cancer HCT-116 control (NT) cells. Interestingly, however, blocking DYRK1A gene expression significantly potentiated the cytotoxic effects of both G1/S targeting drugs, topotecan and cisplatin (left and middle panels). In contrast, blocking DYRK1A expression did not alter the cytotoxic effects of the anti-mitotic inhibitor chemotherapy drug paclitaxel (right panel). The same results were obtained when using two different models of triple negative breast cancer, SUM159 and MDA-MB-231 (Fig. 6b, c). Cytotoxicity and IC50 values are summarized in table S1. To further expand the scope and relevance of our findings, we also assessed DYRK1A inhibition in other cancer models, including lung adenocarcinoma (A459) and the immortalized HeLa cell lines. As shown in (Supplementary Fig. 4) and (Supplementary Table 3), blocking DYRK1A gene expression by means of RNA interference (siRNA) (Supplementary Fig. 4a) significantly potentiated the cytotoxic effects of both G1/S-targeting chemotherapy drugs, topotecan and cisplatin (Supplementary Fig. 4b, c). To further assess the therapeutic values of these results, we then tested combination treatments, using chemotherapy drugs (topotecan and cisplatin) alone or in combination with increasing concentrations of the DYRK1A inhibitor harmine (IC25, IC50). As shown in (Fig. 6d, e) and Supplementary Table 2 increasing concentrations of harmine significantly sensitized cancer cells to topotecan and cisplatin treatments in a concentration-dependent manner, in both colon and TNBC cancer models. Similarly, harmine significantly enhanced drug response to topotecan and cisplatin in HeLa and A549 cells (Supplementary Fig. 4d, e) and Supplementary Table 3. Collectively, these data indicate that specific inhibition of DYRK1A expression or activity strongly potentiates and enhances the cytotoxic effects of G1/S-targeting chemotherapy while not affecting the effects of drugs acting at a later stage, in different models of solid tumors.

**Fig. 7:**
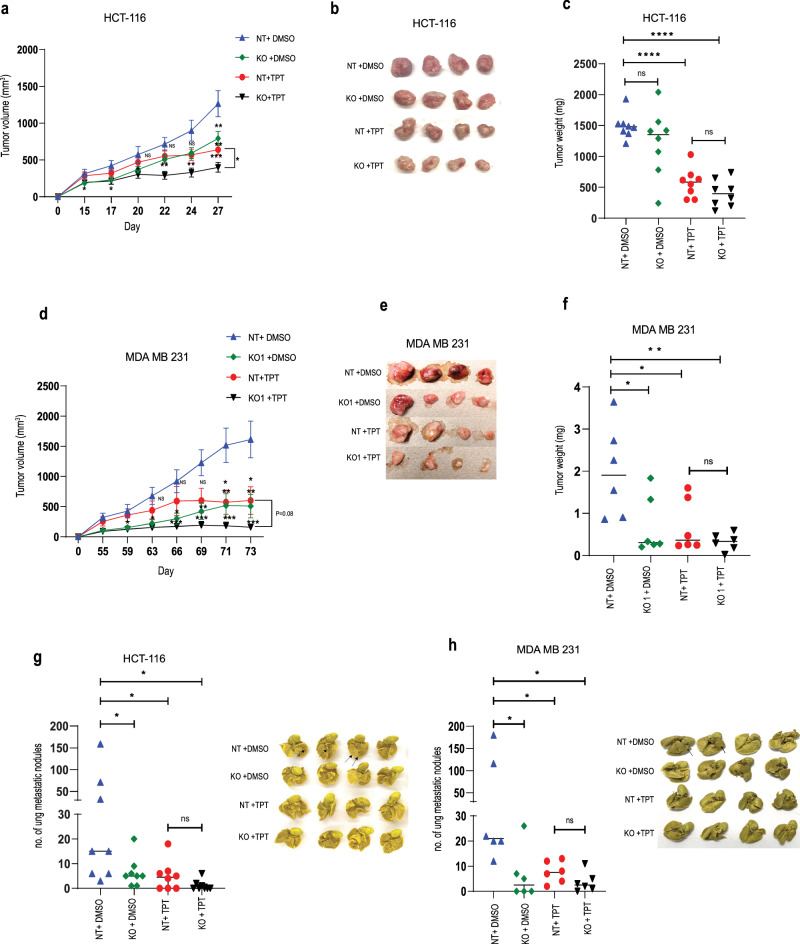
Depletion of DYRK1A sensitizes cancer cells to G1/S phase-targeting chemotherapy drugs *in vivo.* DYRK1A-KO and NT-KO HCT-116 cells were injected (subcutaneously) in male NSG mice (*n* = 8 per group) and treated with topotecan (5 mg/kg) or DMSO bi-weekly. **a** Tumor volumes were measured every 2 days, (**b**, **c**) show representative images of the tumors and dot plots representing ex-in vivo tumor weights, at experimental endpoint. Error bars represent ±SEM of *n* = 8, dot blot middle line represents the. * Represent the *p*-value (*p < 0.05, **p < 0.01, ***p < 0.001, ****p < 0.0001) generated using two-sided *T*-test.DYRK1A-KO and NT-KO MDA MB 231 cells were orthotopically transplanted in the mammary fat pad of NSG mice (*n* = 6 per group) and treated with topotecan (5 mg/kg) or DMSO bi-weekly. **d** Tumor volumes were measured every 2 days, (**e**, **f**) show representative images of the tumors and dot plots representing ex-in vivo tumor weights, at experimental endpoint. Error bars represent ±SEM of *n* = 6, dot blot middle line represents the median. * Represent the *p*-value (*p < 0.05, **p < 0.01, ***p < 0.001, ****p < 0.0001) generated using two-sided *T*-test. **g**, **h** Left panel: Dot plot representing counted lung nodules after fixation with Bouin’s solution in HCT116 and MDA MB 231.Right panel: Representative image showing the lung nodule after fixation. Error bars represent ±SEM of *n* = 8 or *n* = 6, dot blot middle line represents the median. * Represent the *p*-value (*p < 0.05, **p < 0.01, ***p < 0.001, ****p < 0.0001) generated using Mann–Whitney *U*-test (*n* = 8).

Finally, we further tested the ability of DYRK1A inhibition to enhance and promote cancer cells response to chemotherapy treatment, in more clinically relevant settings, using an in vivo colon cancer and TNBC xenograft transplantation models. Briefly, NT and DYRK1A-KO HCT-116 colon cancer cells were transplanted subcutaneously in male NSG mice. For TNBC, NT and DYRK1A-KO MDA-MB-231 cells were transplanted orthotopically into the mammary fat pad in female NSG mice. Once tumors became palpable, animals were injected bi-weekly with topotecan (5 mg/Kg) or vehicle (DMSO). Interestingly, as shown in (Fig. 7a–f), while DYRK1A inhibition or topotecan treatment alone reduced both tumor volume and tumor weight, these effects were greatly enhanced in animals transplanted with DYRK1A-KO cells and further treated with the chemotherapy drug topotecan in both colon and TNBC preclinical in vivo models. Furthermore, HCT-116 colon and MDA-MB-231 cancer cells are highly tumorigenic and can induce the development of spontaneous lung metastasis when used in these xenograft preclinical models. We thus also assessed lung colonization by the cancer cells and quantified the numbers of metastatic lung nodules, following lung resection and Bouin staining. Similar to what observed on primary tumor formation, we found that DYRK1A gene silencing and topotecan treatment alone showed a significant reduction in lung nodule formation. However, these effects were significantly enhanced when topotecan was injected in DYRK1A-KO transplanted animals (Fig. 7g, h). Altogether, these results indicate that DYRK1A specific inhibition efficiently sensitizes tumors to chemotherapy treatment in preclinical colon cancer and TNBC models and suggest that DYRK1A inhibition could prove useful in the clinic if used in combination with G1/S-targeting chemotherapy drug combination treatments.

## Discussion

The function of DYRK1A in cancer has remained multifaceted and controversial. Our findings shed light on the role and contribution of the DYRK1A kinase to human cancer. In this study, we show that DYRK1A acts as a -tumorigenic kinase in two of the most aggressive types of solid tumors, colon cancer and triple negative breast cancer. Our study, importantly, also highlights a new therapeutic strategy, targeting DYRK1A, with promising results to sensitize the tumors to G1/S phase-specific chemotherapy treatment in both triple negative breast cancer and colon cancer. While studies have reported DYRK1A to be implicated in oncogenic pathways such as EGFR stabilization^36^, resisting apoptosis^17^, promoting angiogenesis^37^, and overcoming stress response^38^, others found that DYRK1A could act as a tumor suppressor by inducing quiescence and senescence^28^. Our previous work showed that DYRK1A is upregulated in late tumor stages and its expression is associated with bad prognosis in colorectal cancer. This data will be instrumental to further develop novel therapy based on DYRK1A inhibition to treat patients affected by these incurable cancers. Small molecule DYRK1A inhibitors have been developed by Meijer et al.^39,40^. One promising compound, Leucettinib-21 (LCTB-21) is highly specific to DYRK1A and will enter clinical trials as a treatment plan for cognitive defects associated with Down syndrome^41^. Based on our work, Leucettinib-21 could represent a valuable therapeutic agent in breast and colon cancers, by having the potential for across-disease type activity, thus offering opportunities for rapid drug repositioning and repurposing.

At the molecular level, we found that DYRK1A inhibition releases cells from quiescence to enter and remain in G1/S, preventing or delaying progression towards G2/M phase, further leading to growth arrest both in vitro and in vivo. Our results are consistent with previous reports examining the role of DYRK1A in cell quiescence and G0 entry in glioblastoma^28^ as well as previous studies identifying DYRK1A as a G0/G1 favoring kinase in glioblastoma and normal fibroblasts^9,29^. In agreement with our flow cytometry findings, we found that DYRK1A inhibition leads to increase in expression of G1/S regulators (i.e. Cyclin D1, and CDK4) and a decrease in expression of CENP-A in tumor samples. This is also consistent with a previous study in glioblastoma showing that while DYRK1A inhibition could upregulate cyclin D level it also led to increased p27 and p21 levels, further causing G1 arrest^9^. Altogether, we propose a model in which DYRK1A inhibition leads to accumulation of the cancer cells in the G1/S phase of the cell cycle, delaying/preventing their entry to G2/M, ultimately leading to suppression in cell growth and reduction of the tumor burden in preclinical models of colon and breast cancers.

Using preclinical models of colon cancer in vivo, we also found that DYRK1A inhibition not only led to restriction of tumor growth but also significantly inhibited the spread of the tumors to distant secondary organs such as the lungs, liver and kidney. These findings clearly define DYRK1A as a pro-tumorigenic and pro-metastatic kinase and could explain results from a recent study in which microRNA miR-1246 was found to suppress epithelial-to-mesenchymal transition (EMT) and metastasis by targeting DYRK1A in metastatic breast cancer^42^. Similarly, DYRK1A was found to induce EMT by activating STAT3 and SMAD in hepatocellular carcinoma^43^. Thus, the DYRK1A kinase appears as a critical regulator of metastasis in multiple solid tumor types, highlighting the potential of DYRK1A kinase inhibitors as promising new targeted therapies against metastatic cancers.

Having found that DYRK1A inhibition increases the number of cancer cells in the G1/S phase of the cell cycle, suggesting that DYRK1A inhibition may enhance the efficiency of chemotherapy drugs that act in G1/S. In fact, our data not only show that silencing DYRK1A gene expression potentiates and significantly increases the response of colon and breast cancer cells to G1/S targeting chemotherapy treatments but also highlight the DYRK1A kinase small molecule inhibitor, harmine as a potent chemotherapy sensitizer when used in combination therapy with topotecan and cisplatin. Consistent with our model, these effects are specific to G1/S targeting chemotherapy drugs as DYRK1A inhibition or harmine drug treatment showed no significant effect in promoting or improving chemotherapy drugs targeting post-mitosis phases, such as paclitaxel. This will be critical for selecting proper combinatorial chemotherapy drug treatment when using DYRK1A inhibitors in breast and colon cancer patients. In a clinical context, our results show that targeting DYRK1A kinase reduces the tumorigenesis and the metastatic potential of colon and TNBC cells and sensitizes these cells to the cytotoxic effects of G1/S-targeting anticancer drugs.

A limitation of the study relies on the fact that immunocompromised mice do not completely reflect the natural tumor microenvironment in cancer patients and their response to chemotherapy. Indeed, the use of human cancer cell xenografts to immune incompetent NSG mice neglects the role of tumor infiltrating lymphocytes which are known to play critical roles in chemotherapy response^44,45^. Thus, further preclinical studies using immune reconstituted mice models could prove useful to address the role and contribution of immune cells in this process.

Our results show that DYRK1A inhibition, by means of gene silencing and pharmacological inhibition have strong impact in reducing the metastatic spread of both colon and breast cancer cells from the primary tumor to distant organs. It will be interesting in future studies, to address whether DYRK1A inhibition could also prove useful for treating the progression of metastatic tumors, by assessing the pharmacological inhibition of DYRK1A in already established metastatic tumors.

## Methods

### Drugs and treatments

Topotecan hydrochloride, cisplatin, and paclitaxel were purchased from MedChemExpress (MCE). In vitro, topotecan and paclitaxel were dissolved in DMSO, and Cisplatin was dissolved in 0.9% saline. Harmine was purchased from ABCAM (ab120225) and dissolved in DMSO. For in vivo injection topotecan was prepared as the company recommended, at the day of injection it was dissolved in 10% DMSO, 40% PEG300, 5% Tween-80, and 45% of 0.9% saline.

Harmine IC values were determined for each cell line using 48 h treatments at concentrations ranging from 0.001–50 µM and calculated using GraphPad Prism 8.

### Cell lines and culture conditions

HCT-116, HeLa, and HRK293FT were maintained in Dulbecco’s Modified Eagle Medium (DMEM) (Wisent bio or Sigma Aldrich), A549 and MDA MB 231 were maintained in Roswell Park Memorial Institute (RPMI 1640) (Wisent bio or Sigma Aldrich). Above mentioned cell lines were supplemented with 10% fetal bovine serum (FBS)(Gibco). SUM-159 was maintained in Ham’s F12 media with 5% FBS, 5ug/ml insulin, and 1 ug/ml hydrocortisone (Wisent Bio).

SUM-159 cell line was obtained from Dr. Stephen Ethier. More information about this cell line is available at Breast Cancer Cell Line Knowledge Base (www.sumlineknowledgebase.com).

MDA-MB231 was purchased from ATCC. HEK293FT was obtained from Genhunter. HCT-116, HeLa, and A549 were a gift from the Radiobiology and Experimental Radio-Oncology laboratory, University Cancer Center, Hamburg University, Hamburg, Germany and authenticated using the Powerplex 16HS System. All the cell lines were kept in 37 °C humidified incubator and 5% CO_2_. All cell lines were tested by PCR kit for mycoplasma by Diagnostic Laboratory from Comparative Medicine and Animal Resources Centre (McGill University). All cell lines are mycoplasma negative.

### Generating DYRK1A KO cells using CRISPR-Cas9

DYRK1A gene KO was performed using the LentiCRISPR v2 backbone vector (Addgene plasmid #52961). The cloning procedure was done as described in add gene protocol^46^. The small guide RNA oligo primer sequences designed for DYRK1A KO are shown in Table 1.

**Table 1:** primer sequences for CRISPR-Cas9 knock out cloning

Primer Name	Sequence
DYRK1A_sg1_F	5’-AAACCCTCGGAAATTGGTGTTTCTCAGC-3’
DYRK1A_sg1_R	5’-CACCGCTGAGAAACACCAATTTCCGAGG-3’
DYRK1A_sg2_F	5’-CACCGATGATCGTGTGGAGCAAGAA-3’
DYRK1A_sg2_R	5’-AAACTTCTTGCTCCACACGATCATC-3’
DYRK1A_sg3_F	5’-CACCGTAAAATAATAAAGAACAAGA-3’
DYRK1A_sg3_R	5’-AAACTCTTGTTCTTTATTATTTTAC-3’
DYRK1A_sg4_F	5’-CACCGTGTAAAGGCATATGATCGTG-3’
DYRK1A_sg4_R	5’-AAACCACGATCATATGCCTTTACAC-3’
DYRK1A_sg5_F	5’-CACCGGGTGCAAGCCGAACAGATGA-3’
DYRK1A_sg5_R	5’-AAACTCATCTGTTCGGCTTGCACCC-3’
scsg_F	5′-CACCGCGCTTCCGCGGCCCGTTCAA-3′
scsg_R	5′-AAACTTGAACGGGCCGCGGAAGCGC-3′

The LentiCRISPR v2 vector was digested and dephosphorylated. The digested vector then was purified by the QIAquick Gel Extraction Kit (Qiagen). The pair of oligos for DYRK1A was phosphorylated and annealed using T4 PNK enzyme in a thermocycler by incubating for 30 min at 37 °C and 5 min at 95 °C and ramping down to 25 °C. The annealed oligos were diluted at 1:200 and ligated together with the digested vector using Quick ligase (NEB) for 20 min at room temperature. The cloned vectors were transformed into Stbl3 bacteria (Invitrogen) and streaking it onto an LB agar plate for ampicillin selection. Using the Qiagen plasmid miniprep kit the plasmid Miniprep was prepared. For Lentiviral Production and Infection, the HEK293T was co-transfected with the cloned vector and pMD2. G (Addgene #12259) and psPAX2 (Addgene #12260) as packaging and envelope plasmids. After 24 h, the medium containing the virus was collected by centrifugation at 1200 RPM for 10 min. Viruses in the supernatant were used to infect the cells of interest overnight in the medium with 8 µg/mL of polybrene. Thirty-six hours post-infection, cells were treated for 7 days with puromycin for selection. Efficiency of gene KO was determined by western blotting.

### Small interfering RNA for DYRK1A depletion

Cells were seeded in 6 or 96 well plate and cultured for 16–24 h. Cells were transfected with DYRK1A siRNA or Scrambled siRNA (smart pool, Dharmaco, USA). All the siRNAs were mixed with Opti-MEM® Reduced-Serum Medium and Lipofectamine™ RNAiMAX Transfection Reagent (Invitrogen, USA). The transfection mixture was added to the cells in free antibiotic media and cultured for different time points. Transfection for 48 h was selected for further experiments.

### Publicly available genomic and cancer patient’s datasets

Differential gene expression analysis in Tumor, Normal and Metastatic Tissues TNMplot tool was used to explore the DYRK1A expression in breast cancer compared to normal vs breast cancer tumors and metastatic tissues, we used the TNMplot (https://tnmplot.com/analysis/)^47^. We chose the gene expression comparison and gene chip data then we chose DYRK1A in breast tissue. A Violin plot was selected. Gene Expression database of Normal and Tumor tissues 2 (GENT2) To measure the expression level of DYRK1A in different breast cancer molecular subtypes, we used the GENT2 database (http://gent2.appex.kr/gent2/)^48^. NCBI GEO database that is obtained from microarray platforms (Affymetrix U133A or U133Plus2) was used in this tool^48^. We selected the subtypes tab then we queried DYRK1A.

To further analyze the DYRK1A status in breast cancer, we used the cBioPortal^49,50^. We selected Cancer (METABRIC, Nature 2012 & Nat Commun 2016) (2059 samples) data set. We selected the gene-specific query and chose 2 genomic profile analyses the Mutations (comparing 2059 tumor samples with 548 normal matching samples) and the Putative copy number alterations from DNA copy, then we selected the survival/comparison to observe the clinical characteristic for our query.

The Kmplot tool is a tool used to generate the Kaplan–Meier. We chose breast cancer tissue and searched DYRK1A(211079_s_at) Affymetrix ID, ran RFS analysis and selected Subtype—PAM50 to be shown separately.

### The sulforhodamine B (SRB)

Cells were seeded for 16 to 24 h before the experiment in 96 well plates, at the day of analysis cells were fixed with 50% trichloroacetic acid (TCA) for 1 h at 4 C. Then cells were washed with tap water, dried, and stained with 0.4% sulforhodamine B (SRB) dissolved in 1% acetic acid for 30 min. Plates were washed 3 times with 1% acetic acid and dried. 200 ul/well of 10 mM tris base were added to each well and the absorbance of dye was read at 490 nm by BioTek Gen5 plate reader. Surviving fraction was calculated according to control (DMSO or scrambled infected cells), IC50 was calculated using a sigmoidal curve fitting model using GraphPad prism 8.

### Clonogenic assay

HCT-116 (1000 cells/well) and SUM-159 (500 cells/well) were seeded in 6 well plates and kept in the incubator until colonies were formed. Cells were fixed with 70% ethanol for 30 min washed and stained with crystal violet for 5 min, washed and dried. Colonies with >50 cells/clone were counted manually using a light microscope.

### Cell cycle analysis

For cell cycle synchronization, cells were serum starved for 24 h. Cell cycle was induced through addition of 10% FBS for the indicated periods of time.

For propidium Iodide (PI) staining, cells were centrifuged at low speed and washed them 3x with PBS. 100 ug/ml RNAase was added to 1 million cells for 30 min at 37 °C, then 50 ug/ml PI was added, and cells were visualized by the BD Accuri™ C6 Plus Flow Cytometer.

For visualizing the G0, we performed the Pyronin Y/ Hoechst 33342 double staining. After fixation, cells were washed 3x with PBS and stained with Pyronin Y 2–4 ug/ml and Hoechst 33342 1–2 ug/ml. Cells were analyzed by BD LSRFortessa and gated as described previously^30^.

### Western blot

Cell lysis was performed using SDS lysis buffer (Glycerol, 20% SDS and 1 M Tris, pH 6.8) supplemented with protease inhibitor. Cell lysates were heated for 10 min at 95 °C, sonicated, and centrifuged for 20 min at 15000 RPM. For xenografts, tissue samples were grinded on dry ice and added 50–100 mg of tumor powder in 1 ml lysis buffer (1 M Tris, pH 7.4, 5 M NaCl, 10% Triton. 100 mM EDTA). Whole-cell/xenograft lysate was quantified using the BCA assay kit (Thermo Fisher Scientific).15 or 30 µg of cell lysate was loaded into 8–12% SDS-PAGE gel followed by blotting onto a nitrocellulose membrane (Bio-rad). The membrane was blocked in 5% skimmed milk for 1 h at room temperature, washed with 1X TBS-T buffer, and incubated with the indicated primary antibodies overnight at 4 °C (Anti- DYRK1A (1:1000 dilution, cat# 8765 S, cell signaling technology)), anti- Rb (1:1000 dilution, cat# 9309 L, cell signaling technology), anti- p-Rb (S807/811) (1:1000 dilution, cat# 8516 T, cell signaling technology), anti- cyclin A (1:1000 dilution, cat# 4656, cell signaling technology), anti- cyclin D1 (1:1000 dilution, cat# 2922 S, cell signaling technology), anti- beta-tubulin (1:1000 dilution, cat# 2146 S, cell signaling technology), anti- plk1 (1:1000 dilution, cat# 4513 S, cell Signaling technology), anti- cyclin B1(1:1000 dilution, cat# 4138 S, cell signaling technology), anti- Aurora B (1:1000 dilution, cat# 3094, cell Signaling technology), anti- CENP-A (1:1000 dilution, cat# 2186 S, cell signaling technology), anti- MMP9 (1:1000 dilution, cat# 3852 S, cell signaling technology), anti- snail (1:1000 dilution, cat# 38796 S, cell signaling technology), anti- anti-vimentin (1:1000 dilution, cat# Ab92547, Abcam), anti- cdk4 (1:500 dilution, cat# sc-23896, Santa Cruz biotechnology, anti- beta-actin (1:2000 dilution, cat# A5441, sigma-aldrich)). Secondary antibodies (Anti-rabbit IgG HRP-linked Antibody (1:2000 dilution, cat# 7074 S, cell signaling technology)) or (anti-mouse IgG HRP-linked Antibody (1:2000 dilution, cat# 7076 S, cell signaling technology)) were added for 1 h at room temperature. Chemiluminescence was detected using the ECL kit (Bio-Rad, USA). Protein band quantification was done using Bio-Rad Image Lab software (ChemiDoc™ Touch Gel and Western Blot Imaging System; Bio-Rad). Uncropped blot images are provided in Supplementary Fig. 5.

### In vivo Xenograft studies

All mice were housed and handled following the approved guidelines of the Canadian Council on Animal Care (CCAC) “Guide to the Care and Use of Experimental Animals”. All experiments were performed under the approved McGill University Animal Care protocol (AUP # 7497 to JJL). When experiments reached experimental endpoint, animals were anaesthetized to swiftly render animals unconscious and insensitive to pain. Euthanasia was then induced by quick CO2 release. For surgical procedures, Carprofen (20 mg/kg) was used as an analgesic during the process of implanting cells into the mice mammary fat pads. Tumor tissues were collected and divided into frozen sections and formalin-fixed sections. Lung tissues were put in Bouin’s solution post collection for quantifying lung metastatic nodules. 4 × 10^6^ cells/mouse of HCT-116 NT or DYRK1A KO cells were suspended in DMEM media without serum and injected subcutaneously into the right flank of NSG mice or 600 cells /mouse intravenously in the tail vein of NSG mice. For drug treatment, after reaching a measurable tumor, mice were treated twice per week for 2 weeks with vehicle or topotecan 5 mg/kg.

For SUM-159 and MDA-MB-231 NT or KO, 1 × 10^6^ cells/mouse were suspended in 1:1 ratio of ice-cold PBS and Matrigel and injected into the mammary fat pad. Tumor sizes were measured with a digital electronic caliper three times per week and allowed to reach the maximum volume of 1000 mm^3^ before euthanasia. Tumor volumes were calculated according to the following formula: [4/3 x π x (length/2) × (width/2)2] to generate a growth curve.

### Statistical analysis

Unless otherwise specified, the results are reported as the mean ± SEM of the mean from a minimum of three repeated individual experiments. To assess the variance between groups, a two-sample *T*-test was employed unless otherwise stated, and statistical significance was determined as *P < 0.05.

### Reporting summary

Further information on research design is available in the Nature Research Reporting Summary linked to this article.

### Supplementary information

Supplemental Information

reporting summary

## Data Availability

All publicly available data used in this study were originated from TNMplot (https://tnmplot.com/analysis/), GENT2 database (http://gent2.appex.kr/gent2/), cBioPortal (https://www.cbioportal.org/)^49,50^ we selected Cancer (METABRIC, Nature 2012 & Nat Commun 2016) (2059 samples) data set and Kmplot tool (https://kmplot.com/analysis/).
