# Author Correction: Targeting the DYRK1A kinase prevents cancer progression and metastasis and promotes cancer cells response to G1/S targeting chemotherapy drugs

**DOI:** 10.1038/s41698-024-00628-4

**Published:** 2024-07-08

**Authors:** Amina Jamal Laham, Raafat El-Awady, Maha Saber-Ayad, Ni Wang, Gang Yan, Julien Boudreault, Suhad Ali, Jean-Jacques Lebrun

**Affiliations:** 1 Department of Medicine, Cancer Research Program, McGill University Health Center, Montreal, Quebec H4A 3J1 Canada; 2 College of Medicine, University of Sharjah, Sharjah, 27272 United Arab Emirates; 3 Research Institute for Medical and Health Sciences, University of Sharjah, Sharjah, 27272 United Arab Emirates; 4 College of Pharmacy, University of Sharjah, Sharjah, 27272 United Arab Emirates

**Keywords:** Breast cancer, Colon cancer

Correction to: *npj Precision Oncology* 10.1038/s41698-024-00614-w, published online 05 June 2024

In this article the author name Maha Saber-Ayad was incorrectly written as Maha Saber Ayad. The original article has been corrected.

