# Supplementary material for: Targeting the DYRK1A kinase prevents cancer progression and metastasis and promotes cancer cells response to G1/S targeting chemotherapy drugs
Source: NPJ Precis Oncol. 2024 Jun 5;8:128. doi: 10.1038/s41698-024-00614-w (PMC11153725; doi:10.1038/s41698-024-00614-w)
Supplement: Supplementary file 1 — Supplemental Information [file 41698_2024_614_MOESM1_ESM.pdf]

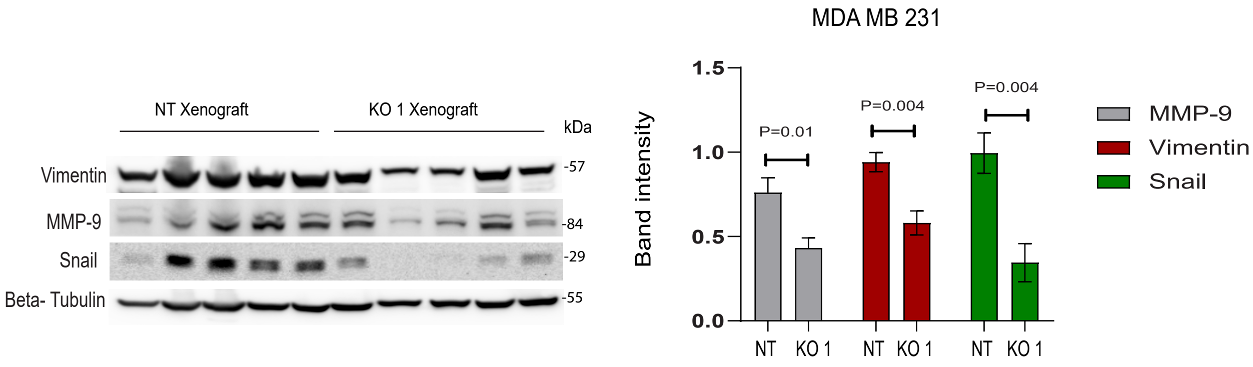


**Supplementary Figure 1. DYRK1A inhibition decreased the expression of mesenchymal markers.** Right panel: Western blot analysis for the expression of mesenchymal markers in DYRK1A-KO and NT MDA MB 231 tumor xenografts. Right panels: Protein expression quantification normalized to beta -tubulin. Error bars represent ±SEM of three independent experiments. The p-values were generated using two-sided T-test.

a

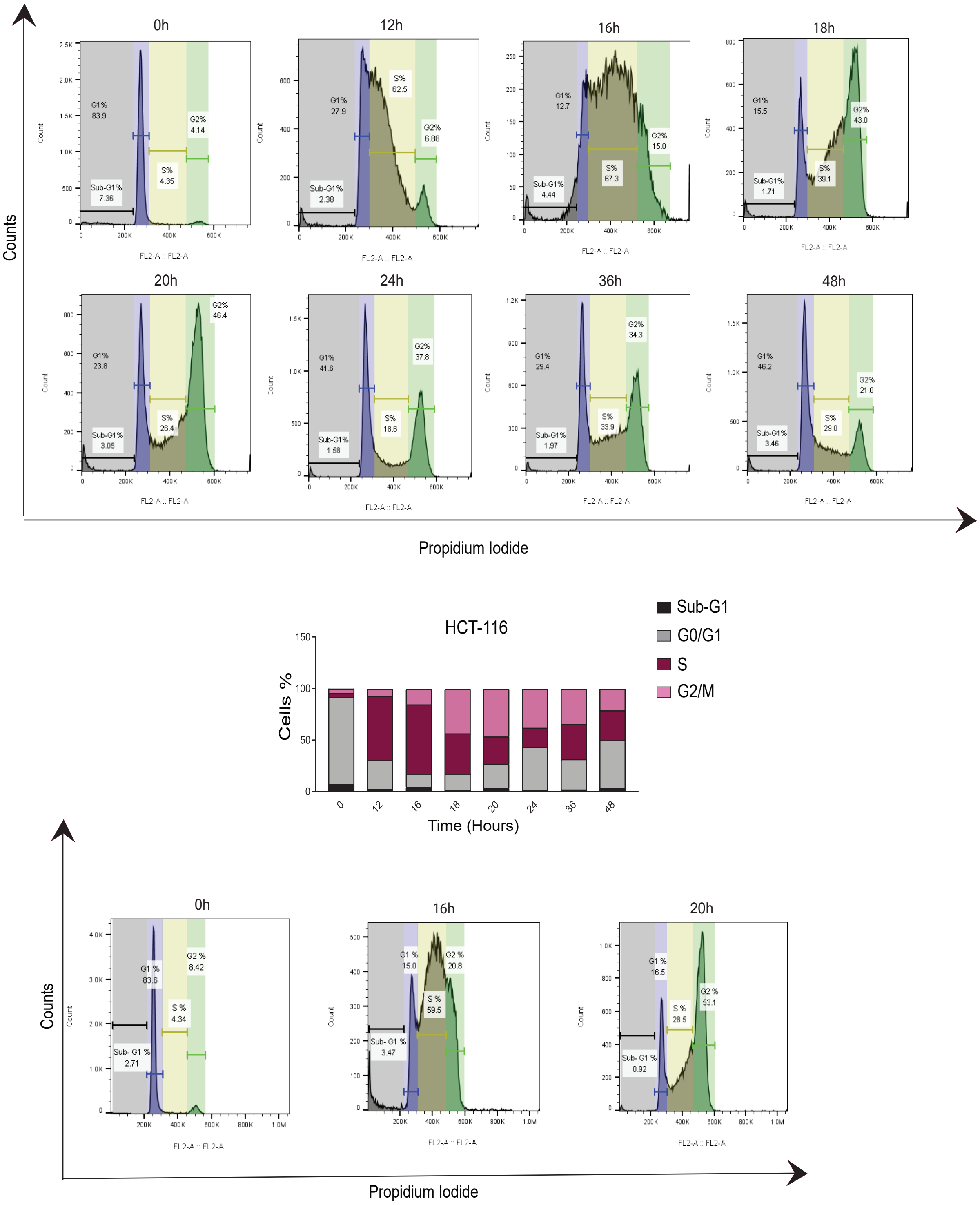

0h
12h
16h
18h
20h
24h
36h
48h
Counts
Propidium Iodide
HCT-116
Cells %
Time (Hours)
Sub-G1
G0/G1
S
G2/M
0h
16h
20h
Counts
Propidium Iodide

**b**

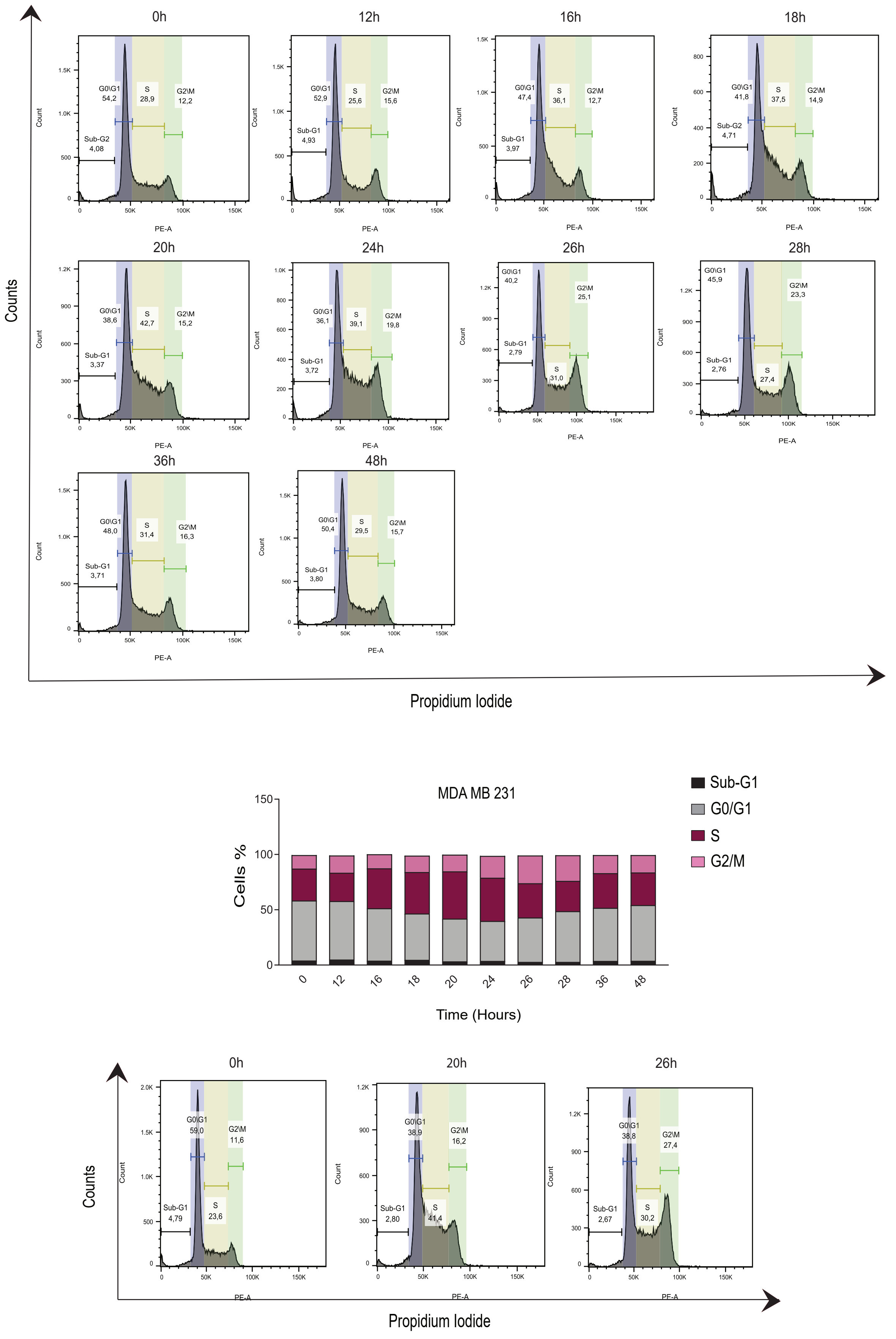
0h
12h
16h
18h
20h
24h
26h
28h
36h
48h
Counts
Propidium Iodide
Count
PE-A
G0\G1 54,2
S 28,9
G2\M 12,2
Sub-G2 4,08
G0\G1 52,9
S 25,6
G2\M 15,6
Sub-G1 4,93
G0\G1 47,4
S 36,1
G2\M 12,7
Sub-G1 3,97
G0\G1 41,8
S 37,5
G2\M 14,9
Sub-G2 4,71
G0\G1 38,6
S 42,7
G2\M 15,2
Sub-G1 3,37
G0\G1 36,1
S 39,1
G2\M 19,8
Sub-G1 3,72
G0\G1 40,2
G2\M 25,1
Sub-G1 2,79
S 31,0
G0\G1 45,9
G2\M 23,3
Sub-G1 2,76
S 27,4
G0\G1 48,0
S 31,4
G2\M 16,3
Sub-G1 3,71
G0\G1 50,4
S 29,5
G2\M 15,7
Sub-G1 3,80
MDA MB 231
Cells %
Time (Hours)
Sub-G1
G0/G1
S
G2/M
G0\G1 59,0
G2\M 11,6
Sub-G1 4,79
S 23,6
G0\G1 38,9
G2\M 16,2
Sub-G1 2,80
S 41,4
G0\G1 38,8
G2\M 27,4
Sub-G1 2,67
S 30,2

**c**

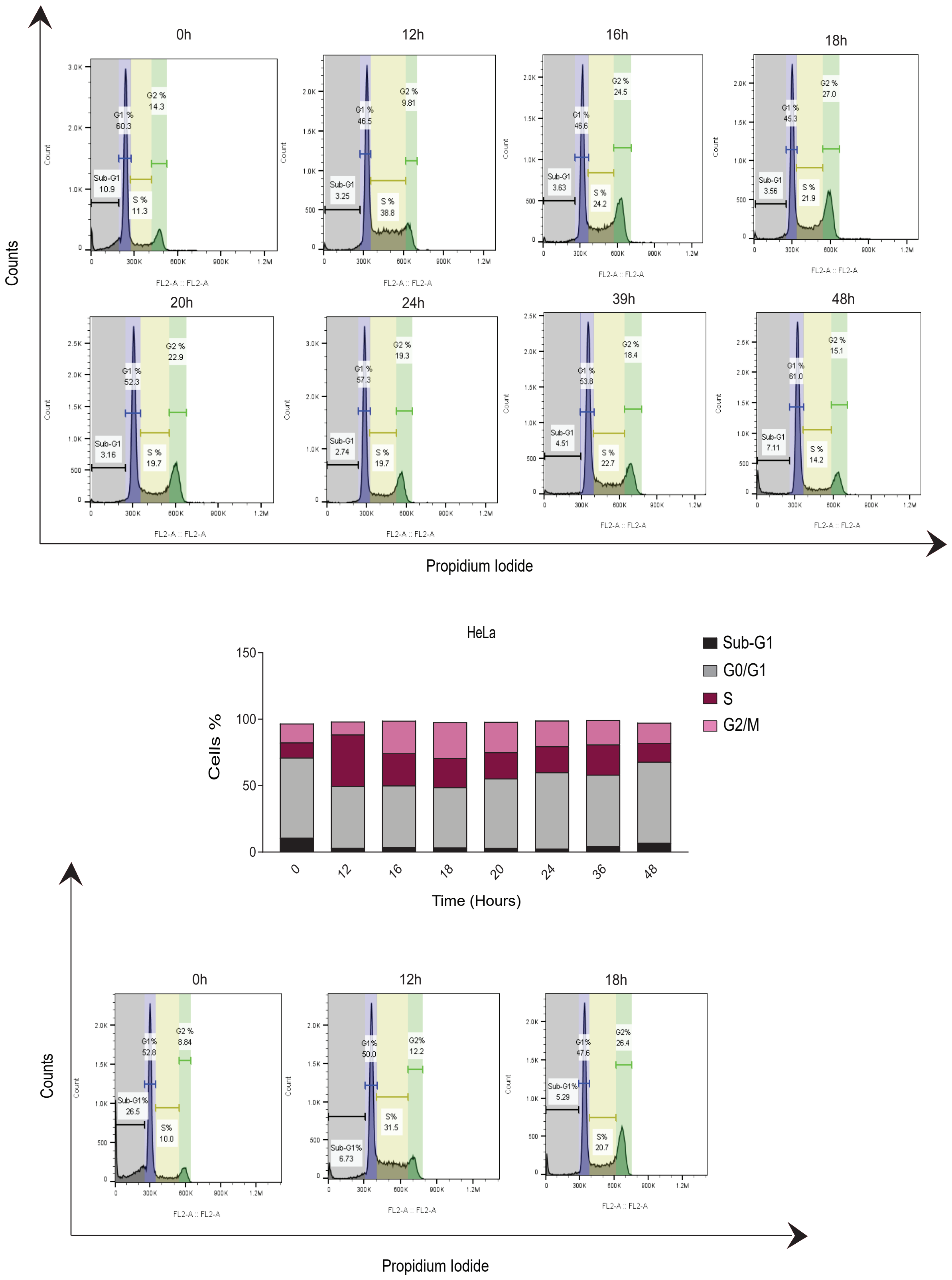


**Supplementary Figure 2. Cell cycle distribution after cell cycle synchronization by serum starvation.**

Upper panels: Histogram represent cell cycle analysis using PI staining for (a)HCT-116, (b) MDA MB 231, and (c) HeLa cells after synchronization by contact inhibition and serum starvation and subsequently allowed to cell cycle through serum addition. Lower panels: Histogram represent cell cycle analysis using PI staining showing the 3 chosen time points for the G0/G1, S, and G2/M phases.

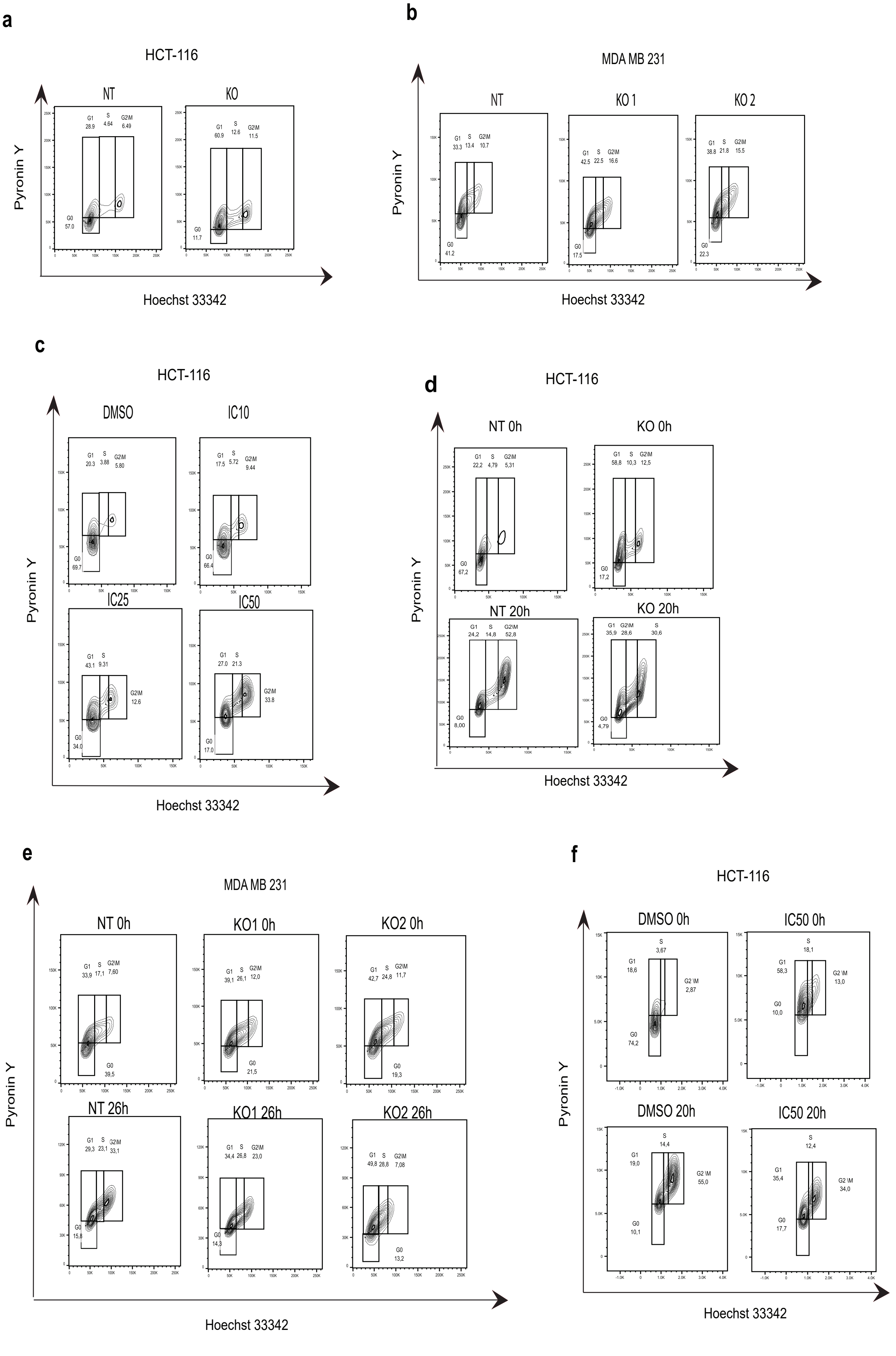


**Supplementary Figure 3. Blocking DYRK1A relieves cancer cells from quiescence, prolonges G1/S and delays G2/M cell cycle entry.** Cell cycle analysis gating for Pyronin Y/Hoechst 33342 double staining for DYRK1A KO and NT-KO in (a) HCT-116 and (b) MDA-MB-231 cells and (c) HCT-116 treated with various concentrations of harmine, as indicated following serum starvation for 24h . Pyronin Y/Hoechst 33342 staining, following serum starvation and release into cell cycle for DYRK1A KO and NT-KO in (d) HCT-116, (e) MDA-MB-231 cells and (f) HCT-116 cells treated with harmine IC50.

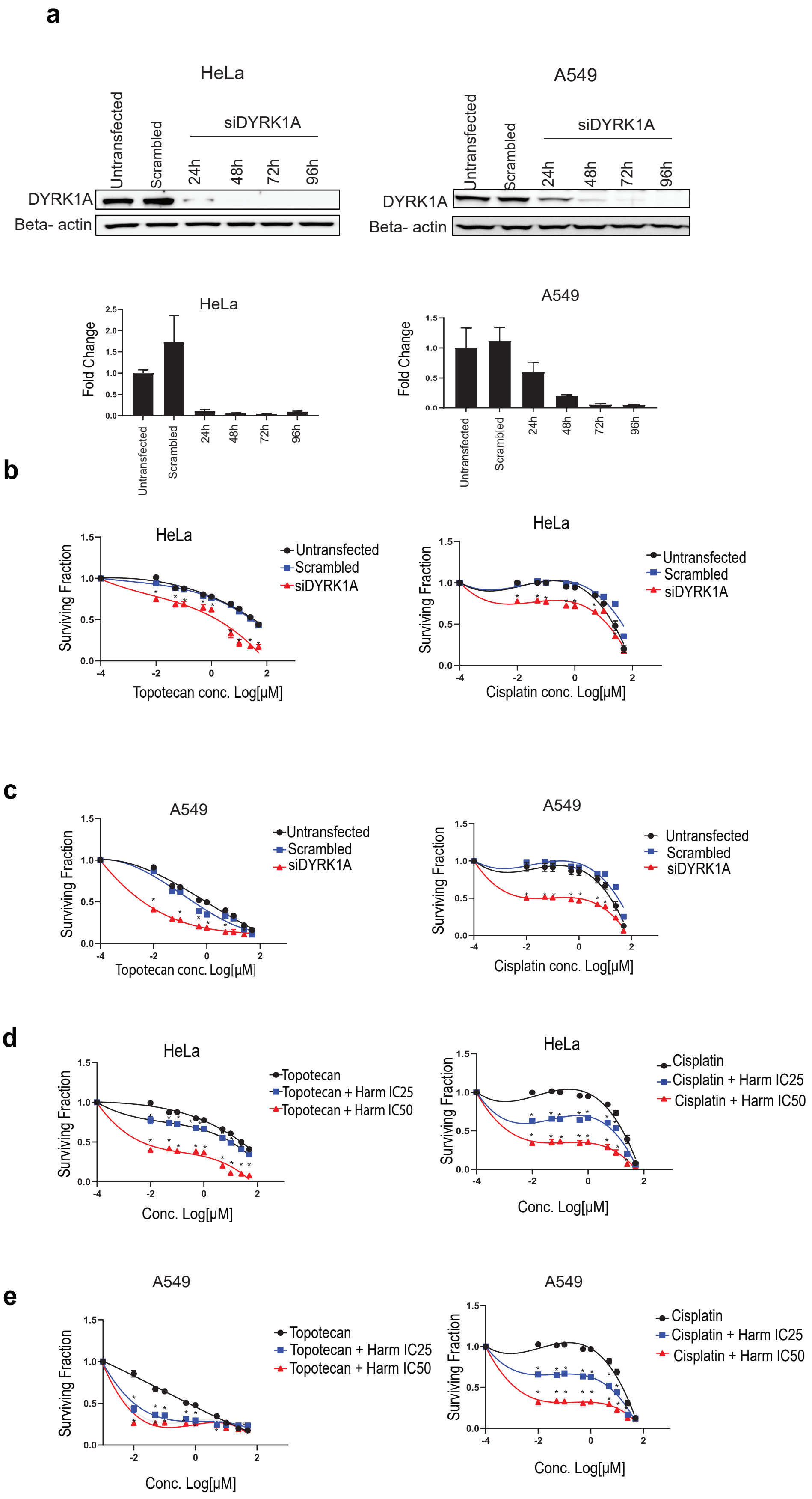


**Supplementary Figure 4. Depletion of DYRK1A enhances cancer cells response to G1 and S-phase chemotherapeutic drugs.** (a) Upper panel: western blot analysis for the DYRK1A expression in A549 and HeLa cells after transfection with siRNA targeting DYRK1A mRNA. Lower panel: Protein expression quantification normalized to beta-actin. Dose-response curves of surviving fraction against various concentrations chemotherapeutic drugs (topotecan and cisplatin, 48hrs treatments) in un-transfected, scrambled siRNA and/or siDYRK1A for (b) HeLa and (c) A549 cells, or parental HeLa(d) and A549 cells (e) co-treated with harmine (IC25 and IC50). Error bars represent ±SEM of three independent experiments. * Represent the p-value (* $p<0.05$, ** $p<0.01$, *** $p<0.001$, **** $p<0.0001$) generated using two-sided T-test.

**a**

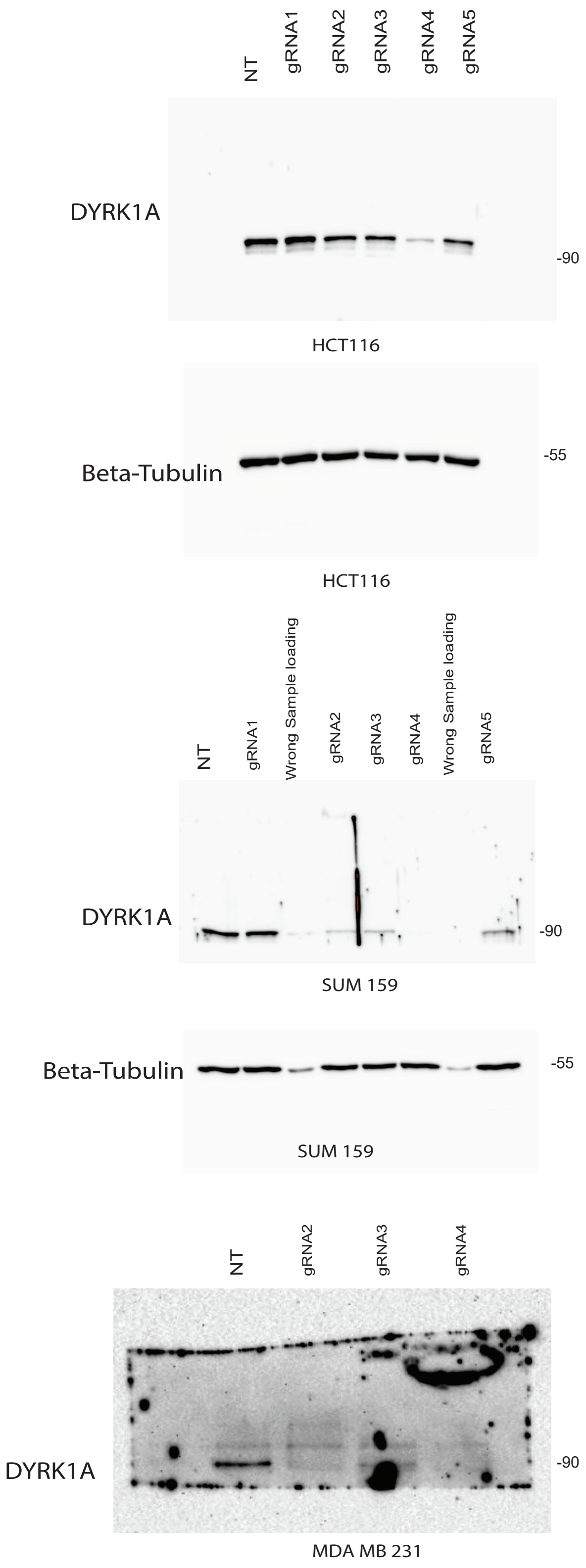
NT
gRNA1
gRNA2
gRNA3
gRNA4
gRNA5
DYRK1A
-90
HCT116
Beta-Tubulin
-55
HCT116
NT
gRNA1
Wrong Sample loading
gRNA2
gRNA3
gRNA4
Wrong Sample loading
gRNA5
DYRK1A
-90
SUM 159
Beta-Tubulin
-55
SUM 159
NT
gRNA2
gRNA3
gRNA4
DYRK1A
-90
MDA MB 231

**b**

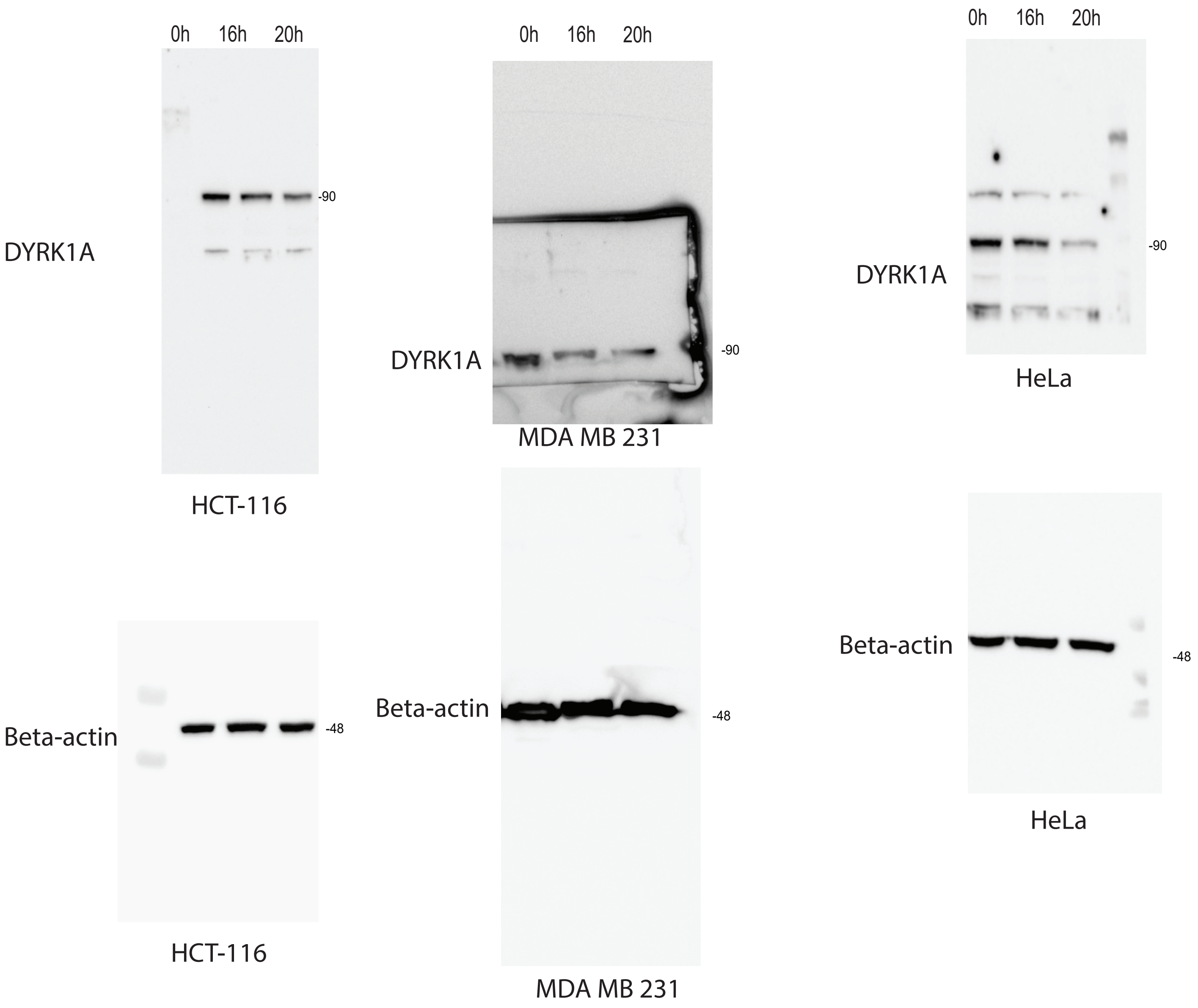
0h
16h
20h
DYRK1A
-90
HCT-116
Beta-actin
-48
HCT-116
0h
16h
20h
DYRK1A
-90
MDA MB 231
Beta-actin
-48
MDA MB 231
0h
16h
20h
DYRK1A
-90
HeLa
Beta-actin
-48
HeLa

d

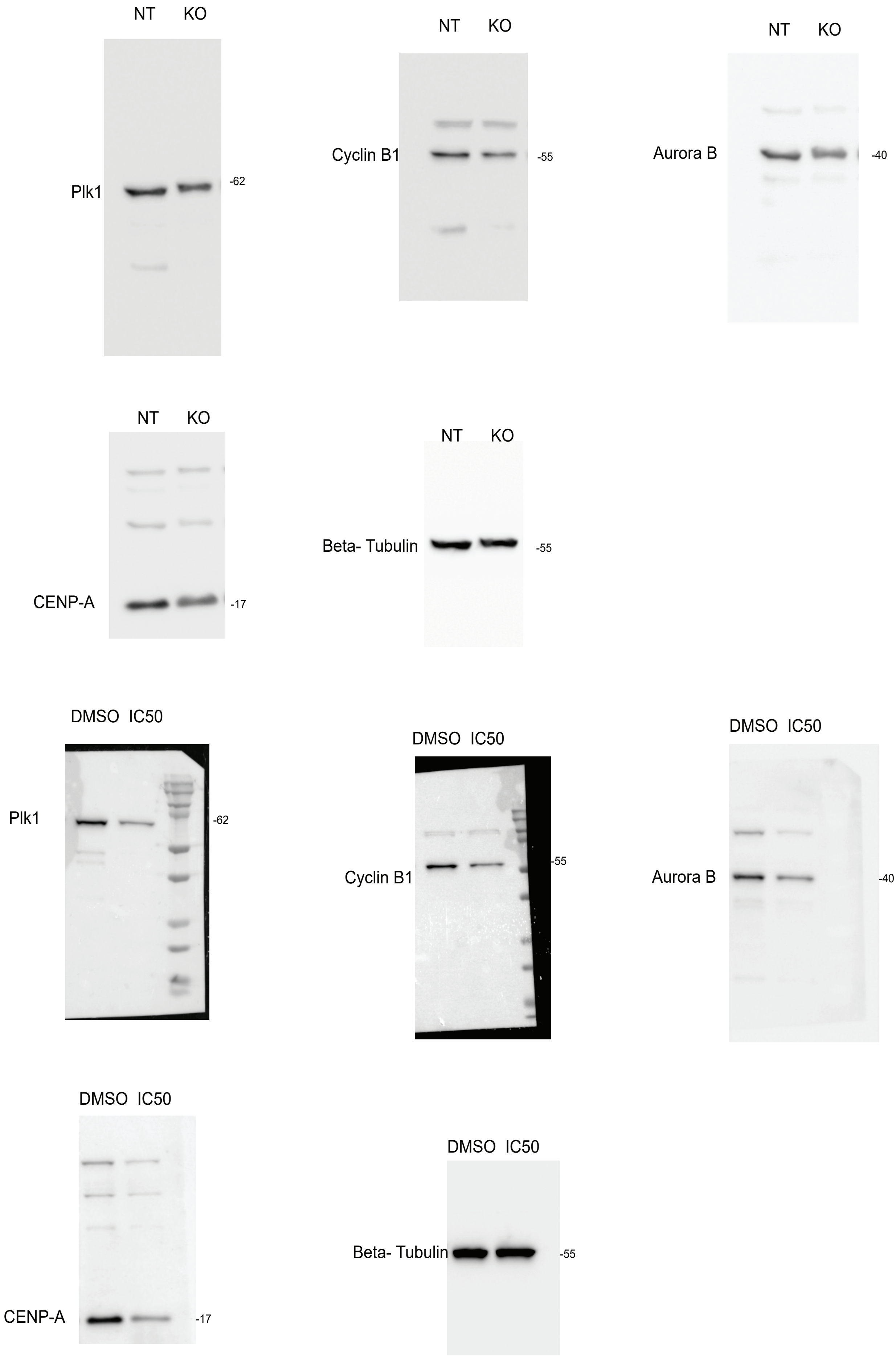
NT KO
Plk1
-62
NT KO
Cyclin B1
-55
NT KO
Aurora B
-40
NT KO
CENP-A
-17
NT KO
Beta- Tubulin
-55
DMSO IC50
Plk1
-62
DMSO IC50
Cyclin B1
-55
DMSO IC50
Aurora B
-40
DMSO IC50
CENP-A
-17
DMSO IC50
Beta- Tubulin
-55

**f**

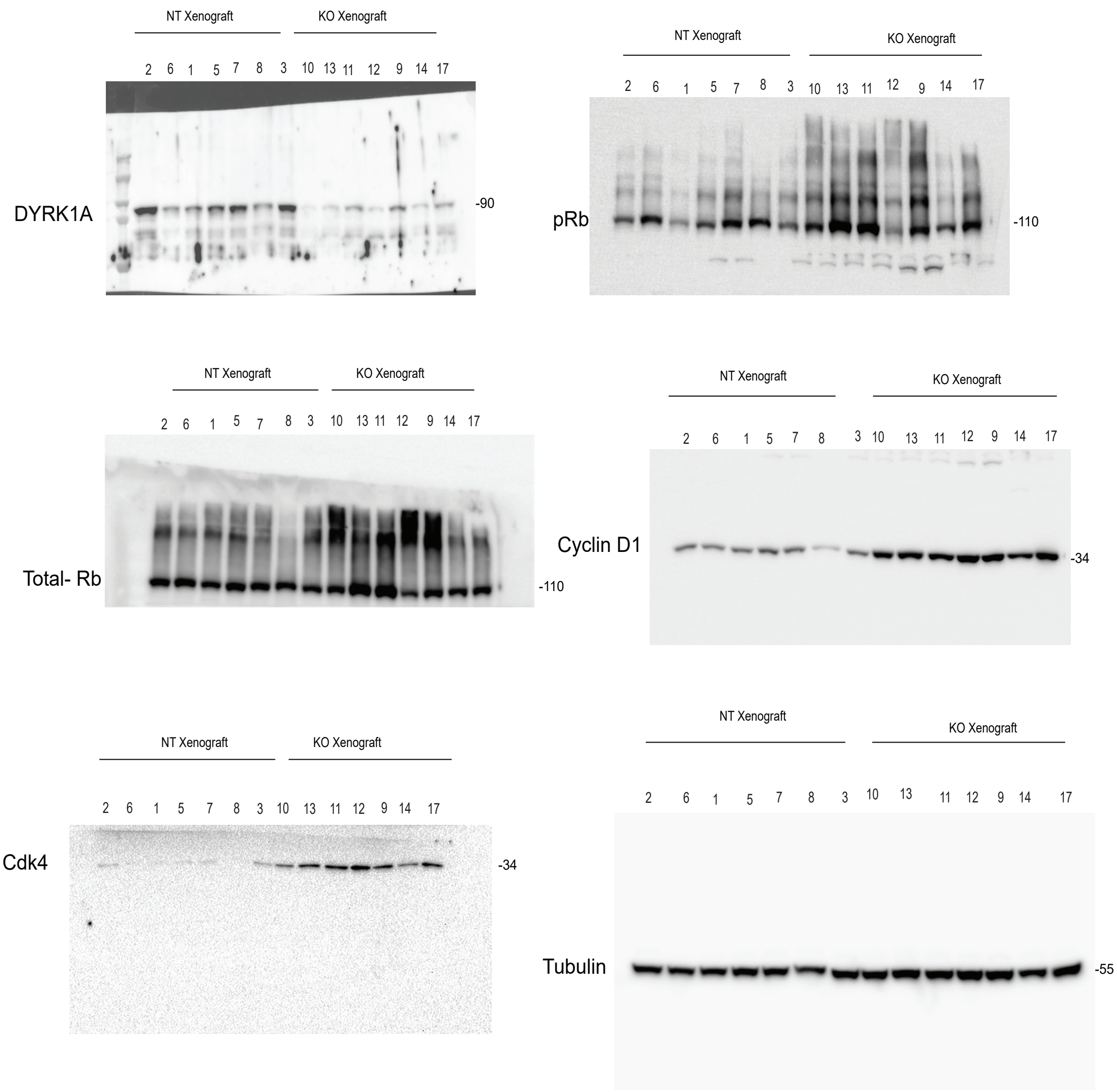
NT Xenograft
KO Xenograft
2 6 1 5 7 8 3 10 13 11 12 9 14 17
DYRK1A
-90
NT Xenograft
KO Xenograft
2 6 1 5 7 8 3 10 13 11 12 9 14 17
pRb
-110
NT Xenograft
KO Xenograft
2 6 1 5 7 8 3 10 13 11 12 9 14 17
Total- Rb
-110
NT Xenograft
KO Xenograft
2 6 1 5 7 8 3 10 13 11 12 9 14 17
Cyclin D1
-34
NT Xenograft
KO Xenograft
2 6 1 5 7 8 3 10 13 11 12 9 14 17
Cdk4
-34
NT Xenograft
KO Xenograft
2 6 1 5 7 8 3 10 13 11 12 9 14 17
Tubulin
-55

f

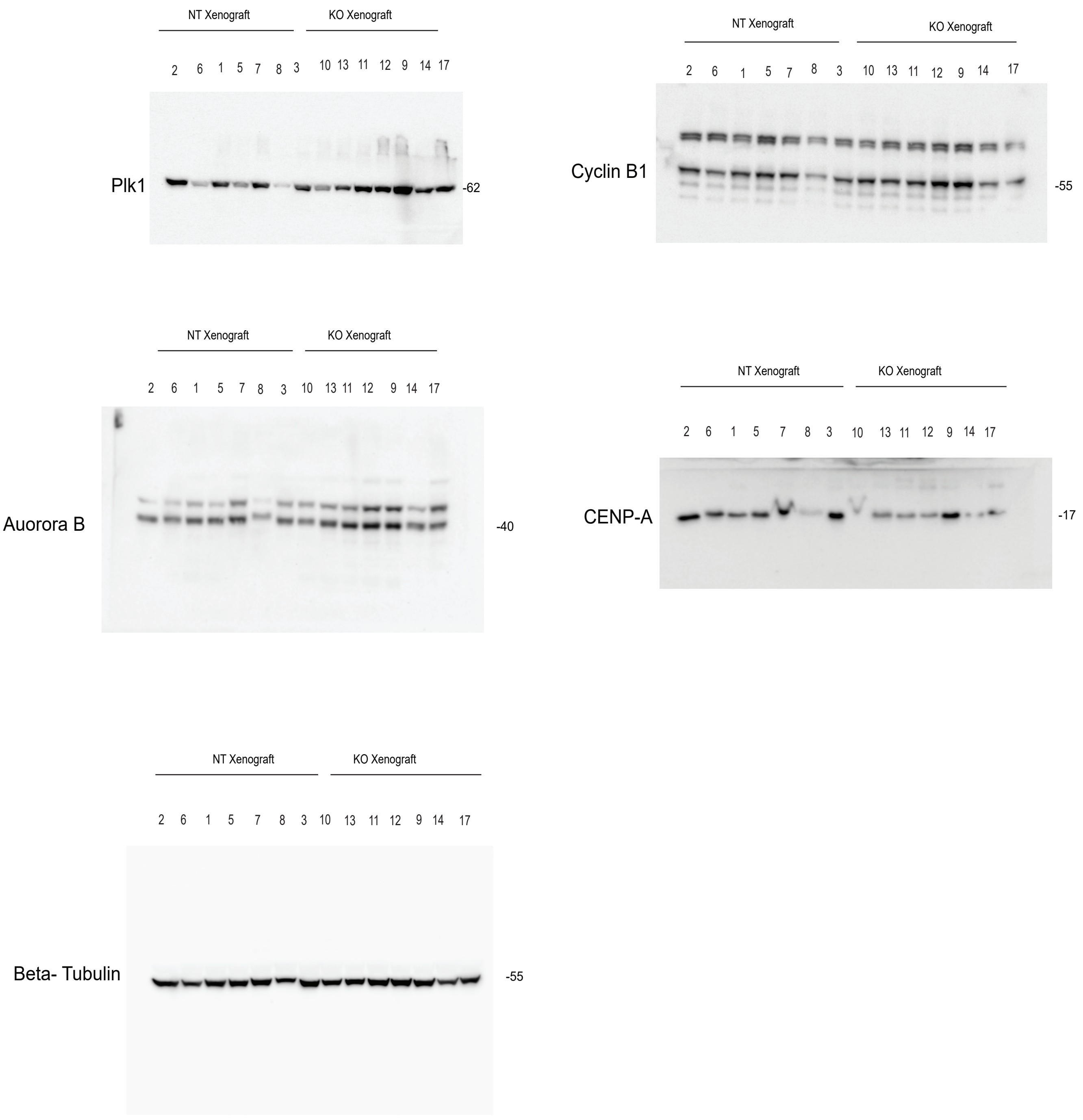


**Supplementary Figure 5. Uncropped western blot membrane image related to the original manuscript.** (a) Related to figure 2 (b) related to figure 3 (c-f) related to figure 5.

**Supplementary table 1.** IC50(μM) ±SEM for topotecan, cisplatin, and Paclitaxel in NT ctrl or DYRK1A KO for HCT-116, SUM-159, and MDA MB 231. IC50 was calculated using a sigmoidal curve fitting model using GraphPad prism 8.

| | IC50(uM) ±SEM | | |
|---|---|---|---|
| | Topotecan | Cisplatin | Paclitaxel |
| HCT-116 | | | |
| NT | 0.34 ± 0.108 | 11.01 ± 0.063 | 0.0127 ± 0.098 |
| KO | 0.020 ± 0.027 | 3.95 ± 0.093 | 0.0129 ± 0.097 |
| SUM-159 | | | |
| NT | 0.47 ± 0.065 | 4.63 ± 0.109 | 0.0279 ± 0.04 |
| KO1 | 0.0093 ± 0.025 | 1.25 ± 0.974 | 0.0126 ± 0.085 |
| KO2 | 0.0056± 0.026 | 1.06 ± 0.900 | 0.0166 ± 0.077 |
| MDA MB 231 | | | |
| NT | 2.31 ± 0.287 | 11.64 ± 0.082 | 〉 50 ± 0.023 |
| KO1 | 0.377 ± 0.082 | 4.46 ± 0.117 | 〉 50 ± 0.092 |
| KO2 | 0.23 ± 0.039 | 2.85 ± 1.59 | 〉 50 ± 0.026 |

**Supplementary table 2.** IC50(μM) ±SEM for topotecan and cisplatin after combination with Harmine IC25 and IC50 for HCT-116 and SUM159. IC50 was calculated using a sigmoidal curve fitting model using GraphPad prism 8.

| | IC50(uM) ±SEM | |
|---|---|---|
| | Topotecan | Cisplatin |
| HCT-116 | | |
| Ctrl | 0.49 ± 0.101 | 23.44 ± 0.02 |
| Harm IC25 | 0.074 ± 0.034 | 7.45 ± 0.092 |
| Harm IC50 | 0.007 ± 0.040 | 0.005 ± 0.041 |
| SUM159 | | |
| Ctrl | 0.14 ±0.009 | 6.11 ±0.09 |
| Harm IC25 | 0.07 ±0.0075 | 5.13 ±0.089 |
| Harm IC50 | 0.01 ±0.024 | 0.024 ±0.034 |

**Supplementary table 3.** IC50(μM) ±SEM for topotecan and cisplatin in parental, scrambled and/or siDYRK1A and/ or after combination with Harmine IC25 and IC50 in A549 and HeLa. IC50 was calculated using a sigmoidal curve fitting model using GraphPad prism 8.

| | IC50(uM) ±SEM | |
|---|---|---|
| | Topotecan | Cisplatin |
| HeLa | | |
| Untransfected | 45.35 ± 0.070 | 49.89 ± 0.018 |
| scrambled | 42.95 ± 0.1 | 28.77 ± 0.051 |
| siDYRK1A | 14.39 ± 0.948 | 1.56 ± 0.025 |
| A549 | | |
| Untransfected | 1.78 ± 0.08 | 29.51 ± 0.015 |
| scrambled | 0.3 ± 0.104 | 30.2 ± 0.049 |
| siDYRK1A | 0.008± 0.031 | 0.023 ± 0.027 |
| HeLa | | |
| Ctrl | 24.9 ± 0.080 | 17.26 ± 0.020 |
| Harm IC25 | 7.57 ± 1.110 | 9.04 ± 0.027 |
| Harm IC50 | 0.012 ± 0.029 | 0.006 ± 0.028 |
| A549 | | |
| Ctrl | 0.85 ± 0.119 | 15.8 ± 0.001 |
| Harm IC25 | 0.03 ± 0.23 | 5.7 ± 0.001 |
| Harm IC50 | 0.005 ± 0.23 | 0.006 ± 0.001 |
